# Effect of short-term exposure to particulate air pollution on heart rate variability in normal-weight and obese adults

**DOI:** 10.1186/s12940-021-00707-0

**Published:** 2021-03-16

**Authors:** Luyi Li, Dayu Hu, Wenlou Zhang, Liyan Cui, Xu Jia, Di Yang, Shan Liu, Furong Deng, Junxiu Liu, Xinbiao Guo

**Affiliations:** 1 Department of Occupational and Environmental Health Sciences, School of Public Health, Peking University, Beijing, 100191 China; 2 Department of Laboratory Medicine, Peking University Third Hospital, Beijing, 100191 China; 3 Department of Otolaryngology Head and Neck Surgery, Peking University Third Hospital, Beijing, 100191 China

**Keywords:** Fine particulate matter, Black carbon, Heart rate variability, Obesity, Circadian rhythm

## Abstract

**Background:**

The adverse effects of particulate air pollution on heart rate variability (HRV) have been reported. However, it remains unclear whether they differ by the weight status as well as between wake and sleep.

**Methods:**

A repeated-measure study was conducted in 97 young adults in Beijing, China, and they were classified by body mass index (BMI) as normal-weight (BMI, 18.5–24.0 kg/m^2^) and obese (BMI ≥ 28.0 kg/m^2^) groups. Personal exposures to fine particulate matter (PM_2.5_) and black carbon (BC) were measured with portable exposure monitors, and the ambient PM_2.5_/BC concentrations were obtained from the fixed monitoring sites near the subjects’ residences. HRV and heart rate (HR) were monitored by 24-h Holter electrocardiography. The study period was divided into waking and sleeping hours according to time-activity diaries. Linear mixed-effects models were used to investigate the effects of PM_2.5_/BC on HRV and HR in both groups during wake and sleep.

**Results:**

The effects of short-term exposure to PM_2.5_/BC on HRV were more pronounced among obese participants. In the normal-weight group, the positive association between personal PM_2.5_/BC exposure and high-frequency power (HF) as well as the ratio of low-frequency power to high-frequency power (LF/HF) was observed during wakefulness. In the obese group, personal PM_2.5_/BC exposure was negatively associated with HF but positively associated with LF/HF during wakefulness, whereas it was negatively correlated to total power and standard deviation of all NN intervals (SDNN) during sleep. An interquartile range (IQR) increase in BC at 2-h moving average was associated with 37.64% (95% confidence interval [CI]: 25.03, 51.51%) increases in LF/HF during wakefulness and associated with 6.28% (95% CI: − 17.26, 6.15%) decreases in SDNN during sleep in obese individuals, and the interaction terms between BC and obesity in LF/HF and SDNN were both statistically significant (*p* <  0.05). The results also suggested that the effects of PM_2.5_/BC exposure on several HRV indices and HR differed in magnitude or direction between wake and sleep.

**Conclusions:**

Short-term exposure to PM_2.5_/BC is associated with HRV and HR, especially in obese individuals. The circadian rhythm of HRV should be considered in future studies when HRV is applied.

**Graphical abstract:**

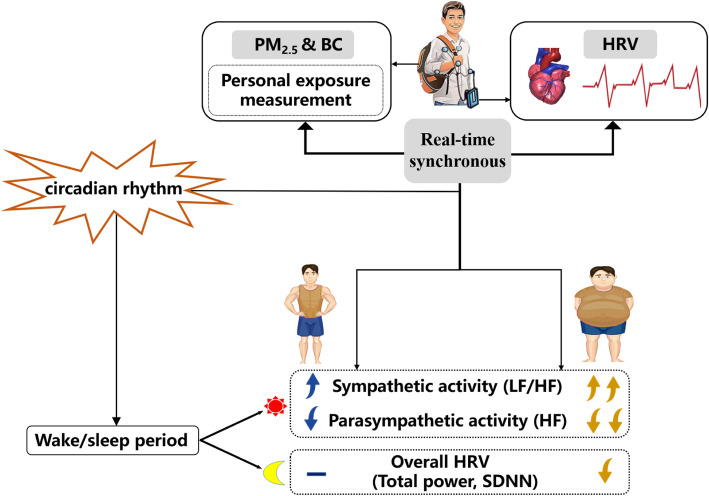

**Supplementary Information:**

The online version contains supplementary material available at 10.1186/s12940-021-00707-0.

## Background

Fine particulate matter (PM_2.5_) pollution has aroused worldwide concern due to its various adverse health effects, particularly in developing countries like China and India where the concentration of particulate matter (PM) is still high [1]. Recently, with the advent of toughest-ever clean air policy in China from 2013 to 2017, the estimated national population weighted annual mean PM_2.5_ concentration decreased from 61.8 to 42.0 μg/m^3^ and the number of PM_2.5_-related excess deaths also decreased by 0.41 million in 2017, which highlighted the effectiveness of China’s clean air actions [2]. However, the association between PM_2.5_ and hospital admission for cardiovascular disease could still be observed even if the daily average PM_2.5_ concentration is below the World Health Organization (WHO) air quality guideline for daily average PM_2.5_ concentration of 25 μg/m^3^ [3]. In addition, as one of the main components of PM_2.5_ and derived from combustion of fossil fuels and biomass burning, black carbon (BC) could be used as an additional index for evaluation of health risks of primary combustion particles and might have a greater impact on cardiovascular system than other particles such as secondary PM [4–7]. Heart rate variability (HRV), a non-invasive marker of cardiac autonomic function, is considered to reflect the interaction between sympathetic activity and parasympathetic activity and the abnormal HRV may be associated with multiple cardiovascular diseases including myocardial infarction, sudden death, coronary artery disease and heart failure [8].

Previous studies have reported that obese individuals are at a higher-risk for cardiovascular diseases. A pooled analysis of 97 prospective cohort studies suggested that for every 5 kg/m^2^ increase in body mass index (BMI), the hazard ratio for coronary heart disease was 1.27 (95% confidence interval [CI]: 1.23, 1.31) [9]. Cardiac autonomic impairment has been observed among the obese with reduction in parasympathetic activity and sympathetic predominance, which might explain the increased prevalence of cardiovascular diseases in obese subjects [10]. Some studies indicated that the association between particulate air pollution and HRV indices was stronger among the obese individuals compared with normal-weight ones [11–14]. Although the existing studies observed that obesity might modify the effect of particulate air pollution on the cardiac autonomic function through stratified analyses, other confounding factors within stratum such as age, health conditions and medication use were not controlled. Thus, further well-designed studies are needed to strengthen these findings.

It should be noted that there is a circadian rhythm for cardiac autonomic nervous system, which is highly significant for every HRV parameter. Some clinical studies have proved that HRV is more affected at night in the patients with Parkinson’s disease and epilepsy [15, 16]. Therefore, it is of great necessity to take the circadian rhythm into consideration to further explore the effects of air pollution on HRV indices. However, most previous studies on particulate air pollution and HRV were conducted during wake or mixed the wakefulness and sleep together.

Moreover, most previous studies used ambient pollutants’ concentrations from the fixed monitoring sites as a proxy for individual exposure to assess the adverse health effects. However, people spend > 80% of their time indoors, so the potential exposure misclassification may emerge when using the ambient pollutant levels and this may have overestimated or underestimated the adverse health effects of air pollutants [17, 18]. A longitudinal study reported an underestimation of the association between ambient PM_2.5_ from air quality monitoring stations and fractional exhaled nitric oxide, whereas our previous work suggested that the outdoor PM data might overestimate its effect on lung function in chronic obstructive pulmonary disease patients [18, 19]. Additional studies are required to determine whether and how this exposure misclassification affects the effects of PM_2.5_/BC on HRV and HR. Therefore, a repeated-measure study was conducted to examine the effects of PM_2.5_/BC exposure on HRV and HR between normal-weight and obese individuals. We hypothesized that the obese individuals might be more susceptible to HRV/HR alterations with PM_2.5_/BC exposure. The study also aimed to investigate whether the above-mentioned effects differed between wake and sleep or between fixed-site and personal exposure measurements. We hypothesized that the above-mentioned effects varied by wake/sleep state and exposure measurements.

## Methods

### Study design and participants

A repeated-measure study was conducted among 97 young (18–26 years) adults from universities close to Peking University Health Science Center (PKU-HSC) from December 2017 to June 2018. The sample size could fulfill the requirements of the present study based on previous experience. Eligibility criteria included: 1) age between 18 and 49 years; 2) with normal sinus rhythm; 3) being normal weight (BMI 18.5–24.0 kg/m^2^) or obese (BMI ≥ 28.0 kg/m^2^). The criterion of BMI was established by the Working Group on Obesity in China from pooled data of Chinese cohorts in order to be more applicable to Chinese adults [20]. Exclusion criteria included: 1) smoking or alcohol drinking; 2) having cardiopulmonary dysfunction, hypertension, diabetes, medication use; 3) unable to complete the entire study procedure. All the participants were divided into the normal-weight group and the obese group based on BMI before the study beginning. In this study, the two groups were comparable for subject numbers, gender and age. A group of 10 subjects were selected for each follow-up visit, of which 5 were normal-weight and 5 were obese to achieve a good match. Participants were asked to keep a time-activity diary to record their wake/sleep periods. The study protocol was approved by the Institutional Review Boards of PKU-HSC (IRB00001052–16066). All participants provided informed consents before participation.

### Exposure measurement

Personal exposure to PM_2.5_ and BC were continually monitored for about 24 h at the same time as HRV measurement and the data were summarized as 5-min averages. PM_2.5_ was measured with a MicroPEM personal exposure monitor (RTI International, USA). BC was measured with a MicroAeth AE51 personal exposure monitor (AethLabs, USA). PM_2.5_ and BC were sampled by the conductive tubing which allowed the sampling ports to be positioned in the breathing zone. During the study period, participants could move freely in their universities such as attending class, resting, and they were required to avoid vigorous physical activity. Each subject was asked to carry the bag containing two portable monitors with them, which were both less bulky and almost without noise. During sleep, participants were asked to put the bag nearby, and to enable the sampling ports closer to the head. Individual noise exposure was measured by a portable noise meter (Model ASV5910; Hangzhouaihua Inc., Hangzhou, CHINA). Real-time temperature and relative humidity (RH) were measured with a MicroPEM personal exposure monitor.

The ambient PM_2.5_ and BC concentrations were also collected. Hourly concentrations of PM_2.5_ were measured in the National Air Quality Control Point within 5 km to the universities where subjects lived, and the minute BC concentrations were measured by an Aethalometer (AE33; Magee Scientific Inc., USA) at a fixed monitoring site in PKU-HSC. All instruments were calibrated before the commencement of the study.

### HRV and HR measurement

During the study, subjects were asked to refrain from caffeine, tea and strenuous physical activities for 24 h before testing. HRV and HR were monitored by ambulatory electrocardiogram (ECG) using a 12-channel Holter recorder (model MGY-H12; DM Software Inc., USA) from 9 am to 9 am the next day. The ECG digital recordings were analyzed by the software (Holter System, version 12.net; DM Software Inc., USA). There were five HRV indices in this study, including one time domain index: standard deviation of all NN intervals (SDNN) and four frequency domain indices: total power (0.01–1.00 Hz), high-frequency power (HF, 0.15–0.40 Hz), low-frequency power (LF, 0.04–0.15 Hz) and the ratio of low-frequency power to high-frequency power (LF/HF). All parameters were calculated in 5-min segments.

### Statistical analysis

Due to skewed distribution (data not shown), the HRV indices and HR were log_10_ transformed before statistical analysis to improve normality and stabilize the variance. To estimate time-lag effects, personal PM_2.5_/BC concentrations were calculated at different moving averages (15 min, 30 min, 1 h, 2 h and 3 h) and then were corresponded to HRV and HR data. Similarly, the ambient concentrations of PM_2.5_ and BC were calculated at different moving averages in hours (1 h, 2 h and 3 h) for further analysis. The exposure windows were chosen by referring to other similar studies, which suggested the significance of shorter lag-phases in future studies [21–23]. Due to repeated measurements on the same subject over time, mixed-effects models were used to estimate the effects of PM_2.5_/BC exposure on HRV and HR.

In the mixed-effects model analysis, PM_2.5_ at different moving averages were utilized as fixed effects, whereas subject and the autocorrelation of HRV were treated as random effects. Other covariates like gender, age, noise (LAeqT), temperature, RH were all included as fixed effects in the model. The model was also applied for BC exposure. To explore whether the effects of PM_2.5_/BC exposure on HRV and HR could be affected by circadian rhythm, the monitoring duration was then divided into waking and sleeping hours according to the time-activity diary. To investigate the effect modification by obesity, which was the primary aim of the study, the effects of PM_2.5_/BC on HRV and HR were compared between the two groups. The interaction terms of PM_2.5_ or BC with obesity were then included into the mixed-effects models [24]. The false discovery rate (FDR) correction was applied to correct for multiple hypothesis tests [25]. Estimated effects were presented as percent changes with their 95% CI in HRV and HR per interquartile range (IQR) increase in PM_2.5_ and BC. The formulae were as follows: [10^(β × IQR)^-1] × 100% and {10^[IQR×(β ± 1.96 × SE)]^-1} × 100%, where β and SE represented the estimated regression coefficient and its standard error, respectively. The smoothed exposure-response curves were fitted for PM_2.5_, BC and 30-min HRV indices [26]. Statistical significance was set as *p* <  0.05. All statistical analyses were performed using R version 3.6.1 (R Foundation for Statistical Computing, Vienna, Austria).

## Results

There were 143 individuals who contacted us in response to recruitment for this study, and 124 met the inclusion/exclusion criteria. One hundred individuals were finally included in the study after a detailed introduction of the whole study, and 97 finished the continuous measurement for about 24 h. Among the 97 participants, 4 were Hui Chinese and the others were Han Chinese. There were 53 subjects (35 males and 18 females) in the normal-weight group and 44 subjects (30 males and 14 females) in the obese group and the average (standard deviation, SD) BMI for the two groups were 21.2 (2.0) kg/m^2^ and 28.8 (1.3) kg/m^2^, respectively. The median ages were 24.0 years and 23.0 years in the normal-weight group and the obese group, respectively (Table S1). Participants were all in good physical condition, without any chronic disease (e.g., cardiovascular diseases and cancer) at baseline. Table 1 showed the distribution of personal PM_2.5_ and BC exposure measurement in 5-min segments as well as noise, temperature and RH data during the study period. The mean (SD) ambient PM_2.5_ and BC were 55.77 (52.72) μg/m^3^ and 2.78 (2.34) μg/m^3^, whereas the personal exposure levels were 28.05 (31.98) μg/m^3^ and 1.80 (2.00) μg/m^3^, respectively. Distributions of 5-min HRV indices and HR were summarized in Table 2.

**Table 1:** Descriptive statistics of PM_2.5_, BC and meteorological factors during the study

Pollutants	N^a^	Mean ± SD	Minimum	25th	Medium	75th	Maximum	IQR
ambient PM_2.5_, μg/m^3^	1220	55.77 ± 52.72	3.00	16.00	40.00	76.00	276.00	60.00
ambient BC, μg/m^3^	1220	2.78 ± 2.34	0.16	0.93	2.10	4.07	10.33	3.14
personal PM_2.5_, μg/m^3^	23,167	28.05 ± 31.98	0.10	8.19	16.04	33.57	272.03	25.38
personal BC, μg/m^3^	23,182	1.80 ± 2.00	0.01	0.45	1.09	2.49	44.05	2.04
Noise (LAeqT), dB	23,185	55.69 ± 5.07	37.54	53.07	55.81	58.23	78.94	5.16
Temperature, °C	23,253	25.98 ± 3.71	9.24	23.84	26.24	28.66	37.16	4.82
Relative humidity, %	23,253	25.28 ± 8.90	6.47	18.69	23.03	30.27	75.60	11.58

*Abbreviations*: *PM*_*2.5*_ fine particulate matter, *BC* black carbon, *SD* standard deviation, *IQR* interquartile range

^a^Observation after removing for missing values and outliers

**Table 2:** Distributions of 5-min heart rate variability (HRV) and heart rate (HR) during the study

HRV indices	All		Normal-weight (*n* = 53)		Obese (*n* = 44)	
	N^a^	Mean ± SD	N^a^	Mean ± SD	N^a^	Mean ± SD
SDNN, ms	40,312	70.4 ± 30.7	20,544	69.2 ± 31.4	19,768	71.3 ± 31.5
Total power, ms^2^	40,312	4807.8 ± 5121.4	20,544	4670.5 ± 5063.1	19,768	4990.9 ± 5447.6
HF, ms^2^	40,312	579.1 ± 669.9	20,544	655.1 ± 776.8	19,768	594.0 ± 641.3
LF, ms^2^	40,312	980.3 ± 742.8	20,544	979.3 ± 770.9	19,768	968.9 ± 753.4
LF/HF	40,312	3.5 ± 3.4	20,544	3.5 ± 3.5	19,768	3.2 ± 3.1
HR, bpm	40,312	73.4 ± 14.9	20,544	74.4 ± 16.0	19,768	70.8 ± 15.2

*Abbreviations*: *SDNN* standard deviation of all normal-to-normal (NN) intervals, *HF* high frequency power, *LF* low frequency power, *LF/HF* ratio of low–high frequency power, *bmp* beats per minute, *SD* standard deviation

^a^ Observation after removing for missing values and outliers

When the waking and sleeping hours were analyzed as a whole, personal exposure to PM_2.5_/BC was associated with a significant decrease in HF and an increase in LF/HF in the obese group, while few significant associations were detected in the normal-weight group. The largest decrease in HF was − 17.01% (95% CI: − 21.83, − 11.90%) with an IQR increase in BC at 3-h moving average, when LF/HF also reached its maximum increase of 21.64% (95% CI: 13.02, 30.92%) in the obese group. Significant decrease in HR was observed in both groups with PM_2.5_ and BC exposure (Table S2).

**Fig. 1:**
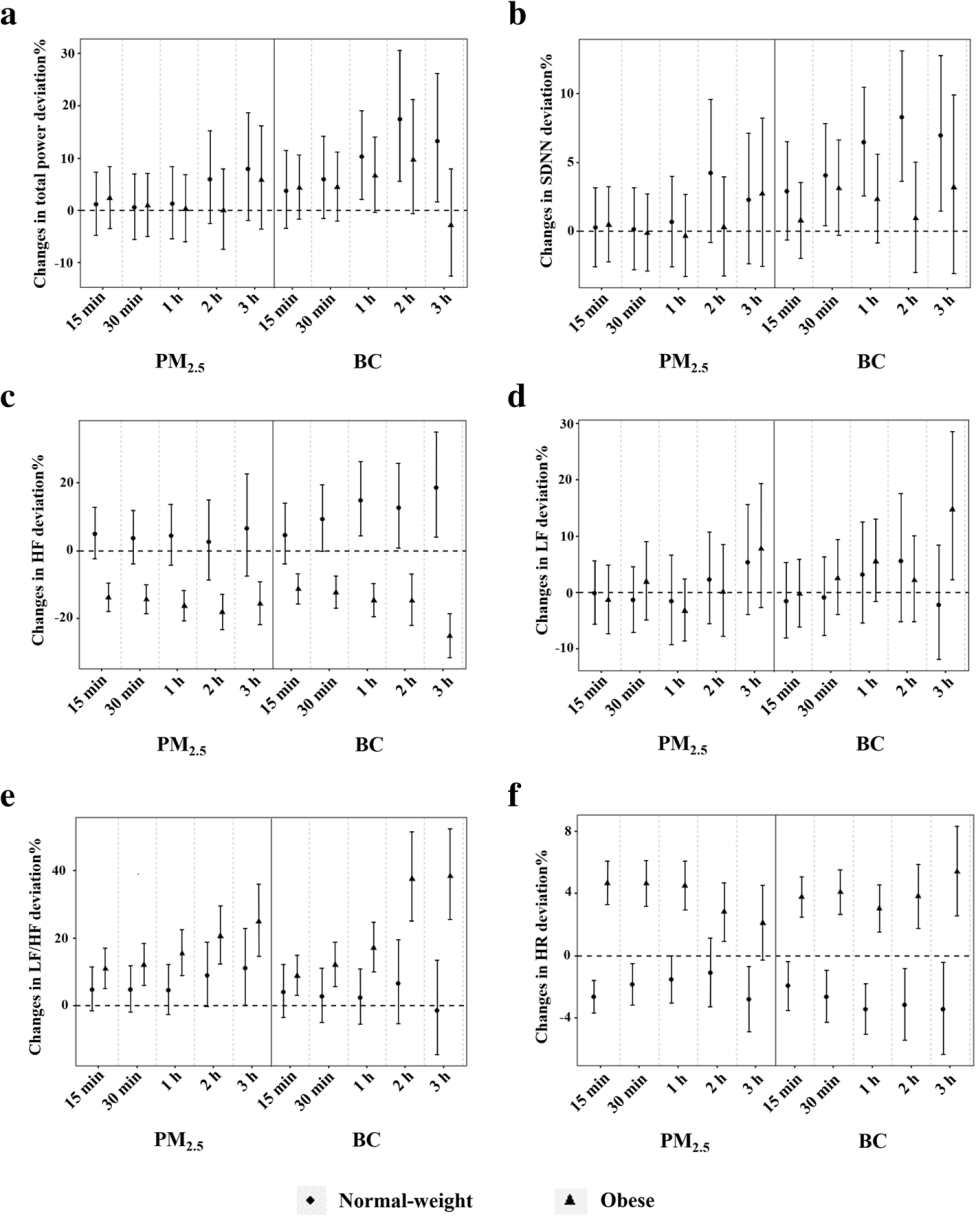
Estimated percent changes with 95% CI in HRV and HR per IQR increase in personal PM_2.5_/BC at different moving averages in the normal-weight and obese groups during wake. **a**, Total power; **b**, SDNN; **c**, HF; D, LF; E, LF/HF; F, HR. CI, confidence intervals; HRV, heart rate variability; HR, heart rate; IQR, interquartile range; PM_2.5_, fine particulate matter; BC, black carbon; SDNN, standard deviation of all normal-to-normal (NN) intervals; HF, high frequency power; LF, low frequency power; LF/HF, ratio of low-frequency power to high-frequency power

When the study period was further divided into waking and sleeping hours, the results indicated that the effects of personal PM_2.5_/BC exposure on HRV and HR were more significant in the obese group, and the effects differed between waking and sleeping hours. In the waking state, the variations in total power and SDNN were similar between the two groups, which suggested an ascendant tendency (Fig. 1a-b). The results also showed that HF was negatively associated with personal PM_2.5_/BC exposure in the obese group, whereas it showed a positive association in the normal-weight group (Fig. 1c). The largest decline in HF in the obese group was − 25.25% (95% CI: − 31.46, − 18.48%) with an IQR increase in BC at 3-h moving average, while an increase of 18.55% (95% CI: 4.03, 35.09%) was observed in the normal-weight group under the same exposure condition (Table S3). LF showed a modest increase with personal PM_2.5_/BC exposure without significant difference between the two groups (Fig. 1d). There was an upward trend of LF/HF in both groups, with a more significant increase in the obese group exposure to PM_2.5_/BC at all moving averages (Fig. 1e). An IQR increase in PM_2.5_ at 1-h moving average was associated with 15.48% (95% CI: 8.80, 22.56%) increases in LF/HF in obese individuals, which was significantly greater than those with normal weight and similar changes were also observed for BC exposure (Table S3). There was a significant increase in HR with personal PM_2.5_/BC exposure in the obese group, whereas the exact opposite was found in the normal-weight group (Fig. 1f).

**Fig. 2:**
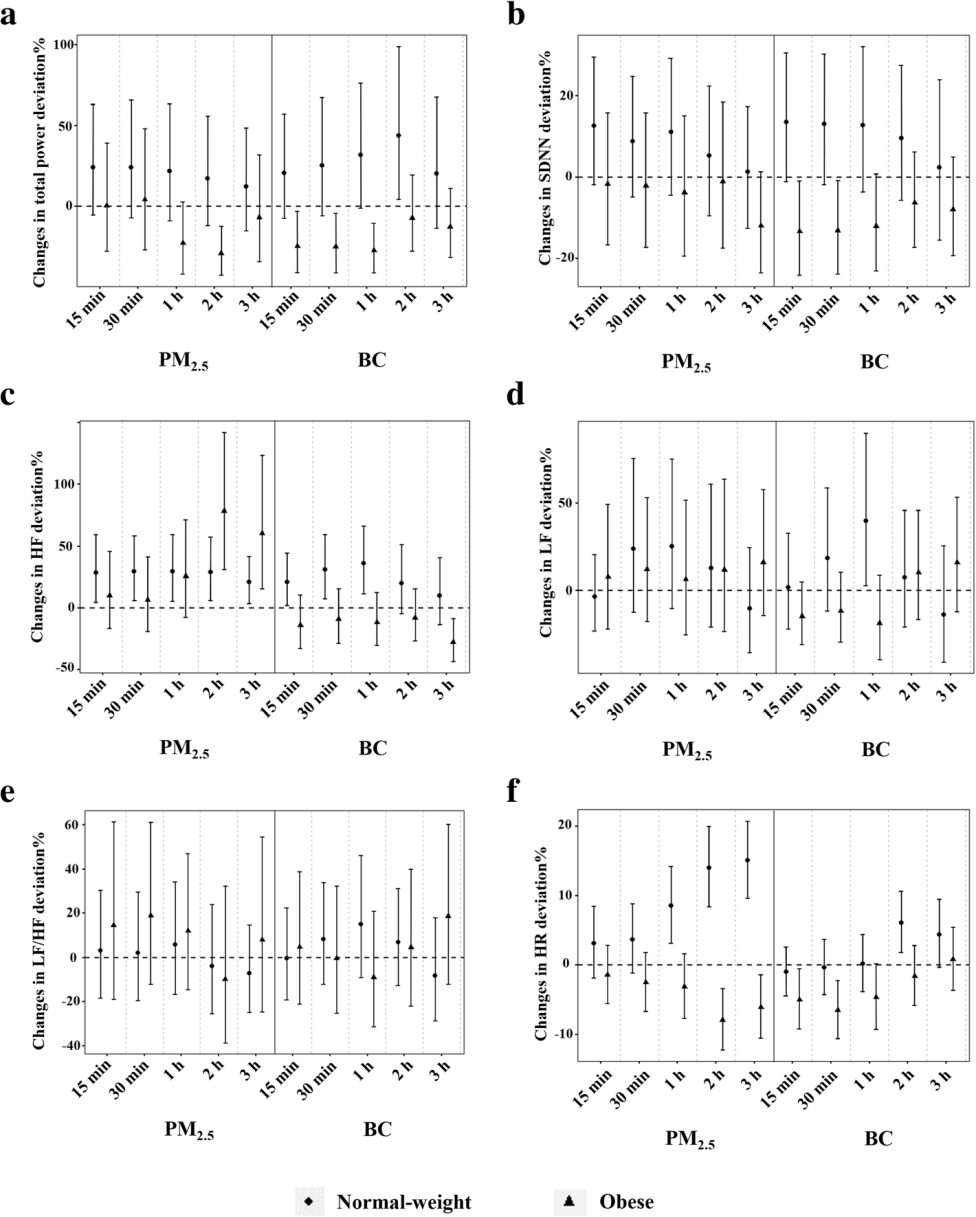
Estimated percent changes with 95% CI in HRV and HR per IQR increase in personal PM_2.5_/BC at different moving averages in the normal-weight and obese groups during sleep. **a**, Total power; **b**, SDNN; C, HF; D, LF; E, LF/HF; F, HR. CI, confidence intervals; HRV, heart rate variability; HR, heart rate; IQR, interquartile range; PM_2.5_, fine particulate matter; BC, black carbon; SDNN, standard deviation of all normal-to-normal (NN) intervals; HF, high frequency power; LF, low frequency power; LF/HF, ratio of low-frequency power to high-frequency power

In the sleep phase, significant decreases in total power and SDNN were found in the obese group with personal PM_2.5_/BC exposure (Fig. 2a-b), and the largest reduction of total power and SDNN were − 28.79% (95% CI: − 42.20, − 12.26%) and − 13.29% (95% CI: − 24.04, − 1.02%) per IQR increase in 2-h PM_2.5_ and 15-min BC moving averages, respectively. However, the corresponding changes were increase of 17.30% (95% CI: − 11.76, 55.94%) in total power and increase of 13.56% (95% CI: − 1.11, 30.41%) in SDNN in the normal-weight group (Table S3). HF, LF and LF/HF changed little with personal PM_2.5_/BC exposure, except for the increase in HF in the normal-weight group (Fig. 2c-e). The changes of HR were opposite between the two groups, which were found to increase in the normal-weight group and to decrease in the obese group (Fig. 2f).

As shown in Table 3, the interactions between obesity and personal PM_2.5_/BC exposure at 2-h moving average on some HRV indices and HR were significant. Similar results were also obtained at other moving averages, which further supported the idea that an interaction between obesity and PM_2.5_ existed and was even more obvious for BC exposure (Table S4–11).

**Table 3:** Estimated percent changes (95% CI) in HRV, HR and *p*-values for interaction term in PM_2.5_/BC-obesity per IQR increase in personal PM_2.5_ and BC at 2-h moving average in normal-weight and obese groups during the waking and sleeping hours

HRV indices	PM_2.5_			BC		
	Normal-weight	Obese	*p*-value for the interaction term (FDR corrected)	Normal-weight	Obese	*p*-value for the interaction term (FDR corrected)
Waking hours						
SDNN	4.28(−0.81, 9.62)	0.30(−3.25, 3.98)	0.216	8.31 (3.67, 13.17)	0.96(−2.99, 5.06)	0.024*
Total power	6.03(−2.47, 15.26)	0.03(−7.36, 7.80)	0.117	17.45 (5.57, 30.67)	9.76(−0.58, 21.18)	0.038*
HF	2.55(−8.50, 14.94)	−18.11(−23.14, −12.75)	0.018^*^	12.55 (0.74,25.74)	−14.79(−21.89, − 7.04)	< 0.001*
LF	2.33(−5.49, 10.79)	0.10(− 7.68, 8.54)	0.269	5.61(−5.15, 17.60)	2.24(−5.10, 10.15)	0.383
LF/HF	8.94(−0.23, 18.94)	20.67 (12.41, 29.54)	0.112	6.43(− 5.27, 19.57)	37.64 (25.03, 51.51)	< 0.001*
HR	−1.10(−3.28, 1.14)	2.81 (0.95, 4.69)	0.010^*^	−3.14(−5.41, −0.82)	3.82 (1.79, 5.89)	< 0.001*
Sleeping hours						
SDNN	5.24(−9.44, 22.31)	−1.12(− 17.45, 18.44)	0.243	9.64(−5.70, 27.48)	−6.28(− 17.26, 6.15)	0.042*
Total power	17.30(−11.76, 55.94)	−28.79(−42.20, − 12.26)	0.176	44.05 (4.33, 98.89)	−6.90(− 27.61, 19.72)	0.041*
HF	28.98 (5.85, 57.17)	78.32 (31.39, 142.01)	0.177	20.10(−4.65, 51.28)	−7.91(−26.64, 15.60)	0.012*
LF	12.72(−21.09, 61.00)	11.78(−23.71, 63.79)	0.368	7.29(−21.17, 46.04)	10.24(−16.73, 45.94)	0.265
LF/HF	−3.79(−25.34, 23.98)	−9.90(− 38.61, 32.24)	0.318	7.01(−12.69, 31.16)	4.62(− 21.83, 40.01)	0.250
HR	14.03 (8.38, 19.98)	−7.89(−12.19, −3.38)	< 0.001*	6.13 (1.84, 10.59)	−1.57(−5.80, 2.85)	0.009*

*Abbreviations*: *CI* confidence intervals, *HRV* heart rate variability, *HR* heart rate, *IQR* interquartile range, *PM*_*2.5*_ fine particulate matter, *BC* black carbon, *SDNN* standard deviation of all normal-to-normal (NN) intervals, *HF* high frequency power, *LF* low frequency power, *LF/HF* ratio of low–high frequency power

^*^ FDR-corrected *p* < 0.05

**Fig. 3:**
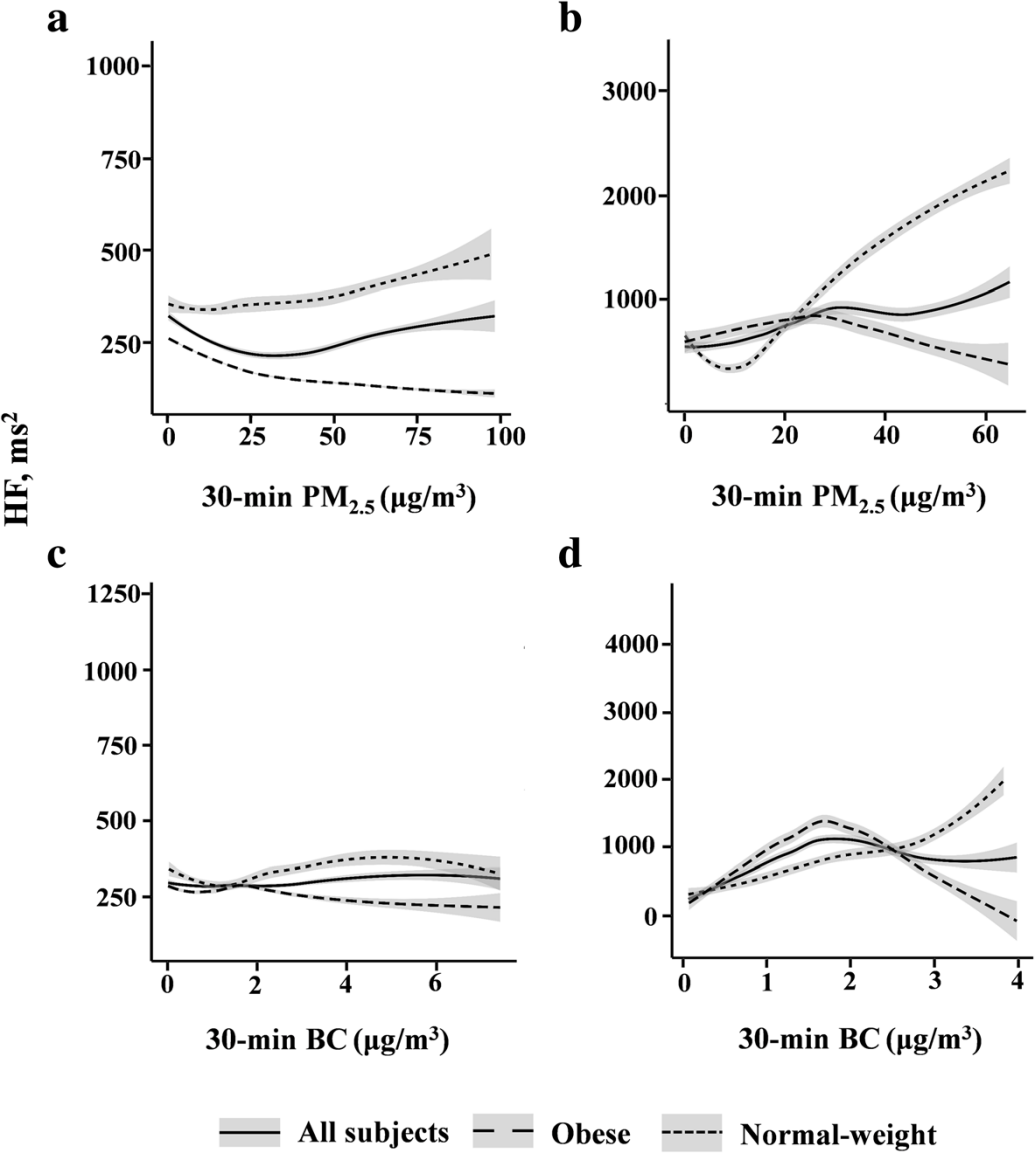
Exposure–response relationship between 30-min personal PM_2.5_ and BC moving average and HF with 95% CI under generalized additive models in all subjects, normal-weight and obese individuals. The degree of freedom was estimated by generalized cross validation. **a** and **c**, waking hours; **b** and **d**, sleeping hours. PM_2.5_, fine particulate matter; BC, black carbon; HF, high frequency power; CI, confidence intervals

As an example, Fig. 3 presented the estimated relationship between personal PM_2.5_/BC exposure at 30-min moving average and HF during wake and sleep. When the whole study population was divided into normal-weight and obese groups, HF showed an increasing trend in the former, while in the latter it started to decrease at relatively lower concentration. As for other HRV parameters, although total power and SDNN increased in both groups in response to the increased PM_2.5_/BC concentration during wakefulness, the levels of total power and SDNN in the obese group were lower and were notably below the overall level. However, during sleep, total power and SDNN in the obese group started to decrease when the concentration of PM_2.5_ or BC reached 40 μg/m^3^ or 3 μg/m^3^. Moreover, LF/HF and HR in the normal-weight group increased smoothly during wakefulness and began to decrease when PM_2.5_ exceeded 50 μg/m^3^, while the obese individuals showed sustained increase (Fig. S1–5).

When the fixed monitoring measurement was used, no significant associations were found between HRV and PM_2.5_/BC exposure in both groups during the whole day (Table S12). After dividing the study period into waking and sleeping hours, only HR had a negative association with ambient PM_2.5_/BC exposure in both groups during wakefulness (Table S13).

## Discussion

In this study, the effects of PM_2.5_/BC exposure on cardiac autonomic nervous system were compared between normal-weight and obese individuals by assessing the real-time personal and ambient PM_2.5_/BC concentrations during waking and sleeping hours. The results suggested that the obese individuals showed greater HRV changes with personal PM_2.5_/BC exposure compared with the normal-weight group, mainly including an increase in sympathetic activity and a decrease in parasympathetic activity. Additionally, this study also confirmed that the effects of PM_2.5_/BC exposure on HRV might be underestimated without considering the circadian rhythm of HRV or using the data from fixed monitoring sites.

When the results were analyzed throughout the day, it supported the hypothesis prior to the present study that the effects of PM_2.5_/BC exposure on HRV were greater in the obese group compared with the normal-weight group, which was reflected by significant decreases in HF and increases in LF/HF. It is known that HF mainly reflects parasympathetic activity and LF/HF is considered to represent sympathovagal balance. The results indicated that personal exposure to PM_2.5_/BC were related to the increased sympathetic activity and reduced parasympathetic activity, which were consistent with previous studies [13, 14]. Although the underlying mechanism remained elusive, some studies provided insights that could possibly explain this. It was suggested that the effects of short-term exposure to air pollution on HRV were more pronounced in those with lower mitochondrial DNA (mtDNA) copy number, and mtDNA copy number was also inversely correlated with BMI, so it was speculated that the discrepancy between the two groups might be related to mtDNA copy number [27–29]. Besides, some studies showed that short-term exposure to air pollution could induce vascular inflammation and the obese individuals might be more prone to vascular inflammation due to pro-inflammatory adipokines and abnormal lipid metabolism [30–32]. Therefore, vascular inflammation might be involved in this phenomenon. The above mechanisms supported our findings to a certain extent, and the underlying mechanisms remained to be further investigated.

On the basis of the above results that there were differences in the effects of personal exposure to PM_2.5_/BC on HRV between the two groups, the study period was further divided into waking and sleeping hours to investigate whether the effects would change between the two states. On the one hand, the results further demonstrated the association between personal exposure to PM_2.5_/BC and disturbed cardiac autonomic nervous function. On the other hand, they suggested that the association differed by wake/sleep state. Specifically, total power and SDNN are the measurements of the overall HRV and they have been found to decrease with air pollution exposure in some studies [12, 13]. However, in the current study, both groups exhibited a small increase in total power and SDNN with PM_2.5_/BC exposure during the waking hours. A few studies reported similar findings to ours, where the subjects were all young adults [26, 33, 34]. Wu et al. proposed that increased SDNN might be a ‘healthy’ response to PM for young adults [35]. Besides, the physiological significance of LF is still controversial, as some studies have argued that LF reflects both sympathetic and parasympathetic activity, while others suggest that LF may reflect baroreflex cardiac effects rather than sympathetic modulation [36, 37]. The present results implied a decrease in HF and an increase in LF/HF, as well as a slight upward trend in LF for obese subjects during the waking hours, which further implied that the existence of negative and positive correlation between personal exposure to PM_2.5_/BC and parasympathetic activity and sympathetic activity, respectively. However, unexpectedly, HF showed a rising trend with BC exposure for normal-weight population during the waking hours. It was demonstrated that when sympathetic pathways were hyperactive or the sympathicotonia became harmful, there would be a counter-regulatory increase in parasympathetic tone accompanied by increased HF, in order to antagonize the sympathetic overactivity and decreased oxygen consumption [38, 39]. Based on these previous studies, our findings raised a hypothesis that stimulus of the external environment (for example, exposure to PM_2.5_/BC) induced the elevated sympathetic activity, which in turn, led to a compensatory of parasympathetic activity manifested as the increased HF with PM_2.5_/BC exposure in young normal-weight individuals. Additionally, the exposure-response relationship showed that compared with the normal-weight group, the obese individuals had a weaker compensatory response and therefore led to the reduced parasympathetic activity, exhibiting only minor or no increase in HF and then followed by a marked decrease with increasing concentration of PM_2.5_/BC. Similarly, LF/HF significantly increased in the obese group compared with the normal-weight group with increasing personal PM_2.5_/BC exposure. These results illustrated that the obese individuals were susceptible to cardiac autonomic dysfunction and presented increased sympathetic activity and decreased parasympathetic activity.

During sleep, personal PM_2.5_/BC exposure were inversely associated with total power and SDNN in the obese group. It should be noted that the decline of SDNN could not only reflect the integral alterations of HRV, but also serves as an independent predictor for heart failure and sudden death [40–42]. According to the exposure-response relationship, total power and SDNN showed the sustained increase in the normal-weight group, which might represent the persistent positive response to PM_2.5_/BC exposure. While the response became much weaker in the obese group especially at higher PM_2.5_/BC concentration evidenced by decreased total power and SDNN. The results further confirmed that the obese individuals were more susceptible to HRV alterations with PM_2.5_/BC exposure especially during sleep. In addition, the above changes of HF and LF/HF during wake with personal PM_2.5_/BC exposure changed in sleep state, which could be due to the circadian rhythm of cardiac autonomic regulation. The activity of HF and LF/HF spectral component had a significant circadian rhythm in healthy subjects. In general, several studies showed that HF increased while LF/HF decreased during the sleeping hours [43, 44]. Thus, with personal PM_2.5_/BC exposure, the increase in HF in the normal-weight group became greater, while the decrease in the obese group became smaller due to the increased activity of HF during sleep. Similarly, the increase in LF/HF in both groups with personal PM_2.5_/BC exposure became nonsignificant due to the decreased activity of LF/HF during sleep. It was also observed that HR decreased among the obese individuals during sleeping hours, which has not been mentioned in previous related studies. Further investigation is needed to determine the cause of the changes.

Although it was not the primary aim of this study, more pronounced interaction terms for BC and obesity compared with PM_2.5_ exposure could be observed, especially in total power and SDNN. The results could be explained, on the one hand, BC was an important component of PM_2.5_ and some studies found that BC might pose a greater risk on human health than other components of PM_2.5_. It could be related to the strong inflammatory response and oxidative stress with BC exposure. On the other hand, several studies noted that compared with frequency domain indices (i.e., HF, LF), short-term exposure to PM_2.5_ had smaller effects on frequency domain indices (i.e., total power, SDNN) [12, 23, 26]. In contrast, SDNN was more sensitive to BC exposure than other HRV indices [6]. Given that both total power and SDNN were the measurements of overall HRV, so it was speculated that they were more sensitive to BC exposure.

The above discussions were based on personal exposure to PM_2.5_/BC, however, by contrasting the effects of personal and ambient PM_2.5_/BC exposure on HRV, it could be found that the association of the latter was weaker than the former both throughout the day and during waking/sleeping hours. From the PM_2.5_ and BC concentrations, higher ambient levels were observed approximately twofold than the personal exposure levels. In this study, anthropogenic activity was an important factor that should be considered. The study participants were all college students and they spent most of their time in dormitories or classrooms according to the time-activity diary. Thus, the fixed-site measurement would overestimate the true exposure levels when subjects stay indoors for long periods, and more importantly, without any typical sources of indoor air pollution (e.g., smoking, cooking, printing). In this case, the effects of PM_2.5_/BC on HRV would be underestimated through fixed-site exposure measurement.

To the best of our knowledge, this is the first study to compare the effects of short-term exposure to PM_2.5_ and BC using personal and fixed-site exposure measurement simultaneously on cardiac autonomic function between normal-weight and obese adults, also considering the circadian rhythm of HRV. This study has several strengths. First, the normal-weight and obese adults were included in the study at the same time and both groups were well matched for subject numbers, gender and age*.* Second, since there was a circadian rhythm of cardiac autonomic activity, comparisons of HRV and HR changes with PM_2.5_/BC exposure were performed between waking and sleeping hours in both groups. Third, the current study used not only the relatively accurate personal exposure to PM_2.5_/BC but also the ambient PM_2.5_/BC levels from fixed monitoring sites to illustrate how could the exposure misclassification by the latter measurement be generated and affect the effects on HRV and HR. However, there remained some limitations with the current study. First, the exposure levels of some gaseous pollutants were not measured, so further research is still needed to identify the impacts of other air pollutants on HRV and HR. Second, all study subjects were young individuals in this study, and they could show better performance in responding to PM_2.5_ and BC exposure, which might limit us to extrapolate the results to some extent. Nevertheless, since the aim of this study was to compare the effects of PM_2.5_ and BC among normal-weight and obese individuals, considering the prevalence of obesity and its related complications would increase with age, the older participants could introduce more confounders [45].

## Conclusions

The study illustrated that the obese adults showed greater HRV changes with short-term exposure to PM_2.5_ and BC than the normal-weight adults, and the effects differed between wake and sleep. The effects of PM_2.5_ and BC on HRV could be underestimated with no distinction for wake/sleep state or only using the data from fixed monitoring sites. This study emphasized that protection should be enhanced especially for the obese individuals and the circadian rhythm of HRV as well as more accurate exposure measurement should be taken into consideration for future research on applications of HRV.

## Supplementary Information

**Additional file 1: Table S1.** General characteristics of the study cohort.** Table S2.** Estimated percent changes in HRV and HR per interquartile range increase in personal PM_2.5_ and BC at different moving averages in the normal-weight (obese) group throughout the day.** Table S3.** Estimated percent changes in HRV indices and HR per interquartile range increase in personal PM_2.5_ and BC at different moving averages in the normal-weight (obese) group during the waking and sleeping hours**. Table S4.** Estimated percent changes (95% confidence intervals) in HRV and HR and *p-*values for interaction term in PM_2.5_-obesity per interquartile range increase in personal PM_2.5_ at 15-min moving average in the normal-weight and the obese groups during the waking and sleeping hours.** Table S5.** Estimated percent changes (95% confidence intervals) in HRV and HR and *p-*values for interaction term in BC-obesity per interquartile range increase in personal BC at 15-min moving average in the normal-weight and the obese groups during the waking and sleeping hours.** Table S6.** Estimated percent changes (95% confidence intervals) in HRV and HR and *p*-values for interaction term in PM_2.5_-obesity per interquartile range increase in personal PM_2.5_ at 30-min moving average in the normal-weight and the obese groups during the waking and sleeping hours.** Table S7.** Estimated percent changes (95% confidence intervals) in HRV and HR and *p*-values for interaction term in BC-obesity per interquartile range increase in personal BC at 30-min moving average in the normal-weight and the obese groups during the waking and sleeping hours.** Table S8.** Estimated percent changes (95% confidence intervals) in HRV and HR and *p*-values for interaction term in PM_2.5_-obesity per interquartile range increase in personal PM_2.5_ at 1-h moving average in the normal-weight and the obese groups during the waking and sleeping hours.** Table S9.** Estimated percent changes (95% confidence intervals) in HRV and HR and *p*-values for interaction term in BC-obesity per interquartile range increase in personal BC at 1-h moving average in the normal-weight and the obese groups during the waking and sleeping hours.** Table S10.** Estimated percent changes (95% confidence intervals) in HRV and HR and p-values for interaction term in PM_2.5_-obesity per interquartile range increase in personal PM_2.5_ at 3-h moving average in the normal-weight and the obese groups during the waking and sleeping hours.** Table S11.** Estimated percent changes (95% confidence intervals) in HRV and HR and p-values for interaction term in BC-obesity per interquartile range increase in personal BC at 3-h moving average in the normal-weight and the obese groups during the waking and sleeping hours.** Table S12.** Estimated percent changes in HRV and HR per interquartile range increase in ambient PM_2.5_ and BC at different moving averages in the normal-weight (obese) group throughout the day. **Table S13.** Estimated percent changes in HRV and HR per interquartile range increase in ambient PM_2.5_ and BC at different moving averages in the normal-weight (obese) group during the waking and sleeping hours.** Figure S1.** Exposure–response relationship between 30-min personal PM_2.5_ and 30-min personal BC moving average and total power with 95% confidence intervals under generalized additive models in all subjects, normal-weight and obese individuals. The degree of freedom was estimated by generalized cross validation. A and C, waking hours; B and D, sleeping hours.** Figure S2.** Exposure–response relationship between 30-min personal PM_2.5_ and 30-min personal BC moving average and SDNN with 95% confidence intervals under generalized additive models in all subjects, normal-weight and obese individuals. The degree of freedom was estimated by generalized cross validation. A and C, waking hours; B and D, sleeping hours.** Figure S3.** Exposure–response relationship between 30-min personal PM_2.5_ and 30-min personal BC moving average and LF with 95% confidence intervals under generalized additive models in all subjects, normal-weight and obese individuals. The degree of freedom was estimated by generalized cross validation. A and C, waking hours; B and D, sleeping hours.** Figure S4.** Exposure–response relationship between 30-min personal PM_2.5_ and 30-min personal BC moving average and LF/HF with 95% confidence intervals under generalized additive models in all subjects, normal-weight and obese individuals. The degree of freedom was estimated by generalized cross validation. A and C, waking hours; B and D, sleeping hours.** Figure S5.** Exposure–response relationship between 30-min personal PM_2.5_ and 30-min personal BC moving average and HR with 95% confidence intervals under generalized additive models in all subjects, normal-weight and obese individuals. The degree of freedom was estimated by generalized cross validation. A and C, waking hours; B and D, sleeping hours.

## Data Availability

Not applicable.

## Abbreviations

PM_2.5_: Fine particulate matter
PM: Particulate matter
WHO: World Health Organization
BC: Black carbon
HRV: Heart rate variability
BMI: Body mass index
HR: Heart rate
RH: Relative humidity
SDNN: Standard deviation of all NN intervals
HF: High-frequency power
LF: Low-frequency power
LF/HF: Ratio of low-frequency power to high-frequency power
IQR: Interquartile range
SD: Standard deviation
CI: Confidence interval
