# Supplementary material for: Effect of short-term exposure to particulate air pollution on heart rate variability in normal-weight and obese adults
Source: Environ Health. 2021 Mar 16;20:29. doi: 10.1186/s12940-021-00707-0 (PMC7968215; doi:10.1186/s12940-021-00707-0)
Supplement: Supplementary file 1 — Additional file 1: Table S1. General characteristics of the study cohort. Table S2. Estimated percent changes in HRV and HR per interquartile range increase in personal PM2.5 and BC at different moving averages in the normal-weight (obese) group throughout the day. Table S3. Estimated percent changes in HRV indices and HR per interquartile range increase in personal PM2.5 and BC at different moving averages in the normal-weight (obese) group during the waking and sleeping hours. Table S4. Estimated percent changes (95% confidence intervals) in HRV and HR and p-values for interaction term in PM2.5-obesity per interquartile range increase in personal PM2.5 at 15-min moving average in the normal-weight and the obese groups during the waking and sleeping hours. Table S5. Estimated percent changes (95% confidence intervals) in HRV and HR and p-values for interaction term in BC-obesity per interquartile range increase in personal BC at 15-min moving average in the normal-weight and the obese groups during the waking and sleeping hours. Table S6. Estimated percent changes (95% confidence intervals) in HRV and HR and p-values for interaction term in PM2.5-obesity per interquartile range increase in personal PM2.5 at 30-min moving average in the normal-weight and the obese groups during the waking and sleeping hours. Table S7. Estimated percent changes (95% confidence intervals) in HRV and HR and p-values for interaction term in BC-obesity per interquartile range increase in personal BC at 30-min moving average in the normal-weight and the obese groups during the waking and sleeping hours. Table S8. Estimated percent changes (95% confidence intervals) in HRV and HR and p-values for interaction term in PM2.5-obesity per interquartile range increase in personal PM2.5 at 1-h moving average in the normal-weight and the obese groups during the waking and sleeping hours. Table S9. Estimated percent changes (95% confidence intervals) in HRV and HR and p-values for interaction term [file 12940_2021_707_MOESM1_ESM.docx]

**Supplementary Material**

**Effect of short-term exposure to particulate air pollution on heart rate variability in normal-weight and obese adults**

Luyi Li, Dayu Hu, Wenlou Zhang, Liyan Cui, Xu Jia, Di Yang, Shan Liu, Furong Deng*, Junxiu Liu*, Xinbiao Guo

**Summary of Contents**

[Table S1. General characteristics of the study cohort 3](#_Toc56869903)

[Table S2. Estimated percent changes in HRV and HR per interquartile range increase in personal PM_2.5_ and BC at different moving averages in the normal-weight (obese) group throughout the day 4](#_Toc56869904)

[Table S3. Estimated percent changes in HRV indices and HR per interquartile range increase in personal PM_2.5_ and BC at different moving averages in the normal-weight (obese) group during the waking and sleeping hours 5](#_Toc56869905)

[Table S4. Estimated percent changes (95% confidence intervals) in HRV and HR and p*-*values for interaction term in PM_2.5_-obesity per interquartile range increase in personal PM_2.5_ at 15-min moving average in the normal-weight and the obese groups during the waking and sleeping hours 6](#_Toc56869906)

[Table S5. Estimated percent changes (95% confidence intervals) in HRV and HR and p*-*values for interaction term in BC-obesity per interquartile range increase in personal BC at 15-min moving average in the normal-weight and the obese groups during the waking and sleeping hours 7](#_Toc56869907)

[Table S6. Estimated percent changes (95% confidence intervals) in HRV and HR and p-values for interaction term in PM_2.5_-obesity per interquartile range increase in personal PM_2.5_ at 30-min moving average in the normal-weight and the obese groups during the waking and sleeping hours 8](#_Toc56869908)

[Table S7. Estimated percent changes (95% confidence intervals) in HRV and HR and p-values for interaction term in BC-obesity per interquartile range increase in personal BC at 30-min moving average in the normal-weight and the obese groups during the waking and sleeping hours 9](#_Toc56869909)

[Table S8. Estimated percent changes (95% confidence intervals) in HRV and HR and p-values for interaction term in PM_2.5_-obesity per interquartile range increase in personal PM_2.5_ at 1-h moving average in the normal-weight and the obese groups during the waking and sleeping hours 10](#_Toc56869910)

[Table S9. Estimated percent changes (95% confidence intervals) in HRV and HR and p-values for interaction term in BC-obesity per interquartile range increase in personal BC at 1-h moving average in the normal-weight and the obese groups during the waking and sleeping hours 11](#_Toc56869911)

[Table S10. Estimated percent changes (95% confidence intervals) in HRV and HR and p-values for interaction term in PM_2.5_-obesity per interquartile range increase in personal PM_2.5_ at 3-h moving average in the normal-weight and the obese groups during the waking and sleeping hours 12](#_Toc56869912)

[Table S11. Estimated percent changes (95% confidence intervals) in HRV and HR and p-values for interaction term in BC-obesity per interquartile range increase in personal BC at 3-h moving average in the normal-weight and the obese groups during the waking and sleeping hours 13](#_Toc56869913)

[Table S12. Estimated percent changes in HRV and HR per interquartile range increase in ambient PM_2.5_ and BC at different moving averages in the normal-weight (obese) group throughout the day.. 14](#_Toc56869914)

[Table S13. Estimated percent changes in HRV and HR per interquartile range increase in ambient PM_2.5_ and BC at different moving averages in the normal-weight (obese) group during the waking and sleeping hours 15](#_Toc56869915)

[Figure S1. Exposure–response relationship between 30-min personal PM_2.5_ and 30-min personal BC moving average and total power with 95% confidence intervals under generalized additive models in all subjects, normal-weight and obese individuals. The degree of freedom was estimated by generalized cross validation. A and C, waking hours; B and D, sleeping hours 16](#_Toc56869916)

[Figure S2. Exposure–response relationship between 30-min personal PM_2.5_ and 30-min personal BC moving average and SDNN with 95% confidence intervals under generalized additive models in all subjects, normal-weight and obese individuals. The degree of freedom was estimated by generalized cross validation. A and C, waking hours; B and D, sleeping hours 17](#_Toc56869917)

[Figure S3. Exposure–response relationship between 30-min personal PM_2.5_ and 30-min personal BC moving average and LF with 95% confidence intervals under generalized additive models in all subjects, normal-weight and obese individuals. The degree of freedom was estimated by generalized cross validation. A and C, waking hours; B and D, sleeping hours 18](#_Toc56869918)

[Figure S4. Exposure–response relationship between 30-min personal PM_2.5_ and 30-min personal BC moving average and LF/HF with 95% confidence intervals under generalized additive models in all subjects, normal-weight and obese individuals. The degree of freedom was estimated by generalized cross validation. A and C, waking hours; B and D, sleeping hours 19](#_Toc56869919)

[Figure S5. Exposure–response relationship between 30-min personal PM_2.5_ and 30-min personal BC moving average and HR with 95% confidence intervals under generalized additive models in all subjects, normal-weight and obese individuals. The degree of freedom was estimated by generalized cross validation. A and C, waking hours; B and D, sleeping hours 20](#_Toc56869920)

**Table S1.** General characteristics of the study cohort

| Variable | Normal-weight (n=53) | Obese (n=44) |
| --- | --- | --- |
| **Age, years** |  |  |
| Mean ± SD | 23.3±1.9 | 23.3±2.0 |
| Median | 24.0 | 23.0 |
| **Gender** |  |  |
| Male | 35 | 30 |
| Female | 18 | 14 |
| **Height, cm** |  |  |
| Mean ± SD | 168.8±0.8 | 168.3±0.7 |
| Median | 168.0 | 167.5 |
| **Weight, kg** |  |  |
| Mean ± SD | 60.6±8.9 | 81.8±7.3 |
| Median | 62.5 | 82.8 |
| **BMI, kg/m^2^** |  |  |
| Mean ± SD | 21.2±2.0 | 28.8±1.3 |
| Median | 21.1 | 28.4 |

Abbreviations: SD, standard deviation; BMI: body Mass index

Table S2. Estimated percent changes in HRV and HR per interquartile range increase in personal PM_2.5_ and BC at different moving averages in the normal-weight (obese) group throughout the day

| Pollutant | Averaging period | HRV | | | | | HR |
| --- | --- | --- | --- | --- | --- | --- | --- |
|  |  | Total power | LF | HF | LF/HF | SDNN |  |
| PM_2.5_ | 15min | 3.01(2.50) | 0.16(0.21) | 0.80(-11.50^***^) | 2.76(9.71^***^) | 0.12(1.63) | -2.05^***^(-1.75^***^) |
|  | 30min | 3.33(0.47) | -1.17(0.68) | 0.59(-12.36^***^) | 2.09(15.54^***^) | -0.04(0.71) | -2.27^***^(-2.20^***^) |
|  | 1h | 4.13(-1.82) | -1.41(1.31) | 2.60(-13.75^***^) | 1.08(11.91^***^) | -0.02(-0.36) | -2.72^***^(-3.03^***^) |
|  | 2h | 1.71(-3.12) | -4.67(5.00) | 1.40(-14.87^***^) | -1.39(16.21^***^) | -2.24(-0.69) | -2.87^***^(-4.33^***^) |
|  | 3h | -1.27(-0.45) | -9.37^*^(10.53^***^) | -0.04(-13.84^***^) | -3.11(20.93^***^) | -3.37(0.35) | -3.44^***^(-5.05^***^) |
| BC | 15min | 4.08(2.64) | -0.62(0.53) | 3.85(-6.59^***^) | 4.87(2.73) | 1.84(0.60) | -2.43^***^(-0.99) |
|  | 30min | 4.26(1.79) | -1.33(1.48) | 6.27^*^(-7.09^***^) | 3.08(4.77) | 2.45(0.98) | -3.55^***^(-1.42^*^) |
|  | 1h | 5.51(3.38) | -3.70(2.85) | 8.05^*^(-10.32^***^) | 3.98(8.45^*^) | 2.74(1.85) | -4.23^***^(-1.86^***^) |
|  | 2h | 8.08(1.57) | -10.22^*^(9.45^***^) | 4.13(-14.43^***^) | -2.26(7.72^*^) | 3.80(0.49) | -4.76^***^(-2.59^***^) |
|  | 3h | 2.35(1.23) | -18.00^***^(12.36^***^) | 1.63(-17.01^***^) | -9.60(21.64^***^) | 0.28(0.38) | -5.92^***^(-2.97^***^) |

Abbreviations: HRV, heart rate variability; HR, heart rate; PM_2.5_, fine particulate matter; BC, black carbon; LF, low frequency power; HF, high frequency power; LF/HF, ratio of low–high frequency power; SDNN, standard deviation of all normal-to-normal (NN) intervals.

^*^ FDR-corrected p < 0.05; ^***^ FDR-corrected p < 0.01.

Table S3. Estimated percent changes in HRV indices and HR per interquartile range increase in personal PM_2.5_ and BC at different moving averages in the normal-weight (obese) group during the waking and sleeping hours

|  | Pollutant | Averaging period | HRV | | | | | HR |
| --- | --- | --- | --- | --- | --- | --- | --- | --- |
|  |  |  | Total power | LF | HF | LF/HF | SDNN |  |
| Waking hours | PM_2.5_ | 15min | 1.13(2.33) | -0.08(-1.32) | 5.04(-13.80^***^) | 4.72(10.88^***^) | 0.27(0.49) | -2.65^***^(4.66^***^) |
|  |  | 30min | 0.60(0.92) | -1.32(1.93) | 3.70(-14.34^***^) | 4.69(12.06^***^) | 0.14(-0.13) | -1.84^*^(4.64^***^) |
|  |  | 1h | 1.32(0.29) | -1.54(-3.23) | 4.36(-16.28^***^) | 4.51(15.48^***^) | 0.67(-0.33) | -1.51(4.51^***^) |
|  |  | 2h | 6.03(0.03) | 2.33(0.10) | 2.55(-18.11^***^) | 8.94(20.67^***^) | 4.28(0.30) | -1.10(2.81^*^) |
|  |  | 3h | 7.97(5.84) | 5.44(7.80) | 6.55(-15.67^***^) | 10.99(24.96^***^) | 2.30(2.74) | -2.80(2.11) |
|  | BC | 15min | 3.77(4.32) | -1.57(-0.21) | 4.69(-11.33^***^) | 4.01(8.79^*^) | 2.90(0.79) | -1.94(3.77^***^) |
|  |  | 30min | 6.00(4.44) | -0.82(2.56) | 9.28(-12.33^***^) | 2.75(12.04^***^) | 4.08(3.14) | -2.62^*^(4.08^***^) |
|  |  | 1h | 10.31^*^(6.66) | 3.23(5.54) | 14.83^*^(-14.63^***^) | 2.29(17.19^***^) | 6.49^***^(2.34) | -3.42^***^ (3.03^***^) |
|  |  | 2h | 17.45^*^(9.76) | 5.61(2.24) | 12.55(-14.79^***^) | 6.43(37.64^***^) | 8.31^***^(0.96) | -3.14^*^(3.82^***^) |
|  |  | 3h | 13.21(-2.81) | -2.19(14.74) | 18.55^*^(-25.25^***^) | -1.58(38.43^***^) | 6.99^*^(3.21) | -3.42 (5.40^***^) |
| Sleeping hours | PM_2.5_ | 15min | 24.30(0.65) | -3.76(7.77) | 28.83(10.16) | 3.14(14.53) | 12.66(-1.73) | 3.17(-1.41) |
|  |  | 30min | 24.34(4.31) | 23.8(11.96) | 29.47(6.75) | 2.17(19.01) | 8.82(-2.11) | 3.70(-2.50) |
|  |  | 1h | 22.20(-22.47) | 25.10(6.27) | 29.55(25.83) | 5.81(12.17) | 11.02(-3.75) | 8.56^***^(-3.11) |
|  |  | 2h | 17.30(-28.79^***^) | 12.72(11.78) | 28.98(78.32^***^) | -3.79(-9.90) | 5.24(-1.12) | 14.03^***^(-7.89^***^) |
|  |  | 3h | 12.57(-6.60) | -10.60(16.17) | 20.97(60.52^*^) | -7.08(8.03) | 1.36(-11.94) | 15.04^***^(-6.08) |
|  | BC | 15min | 20.81(-24.30) | 1.64(-15.01) | 21.29(-13.78) | -0.49(4.80) | 13.56(-13.29) | -0.95(-4.97) |
|  |  | 30min | 25.54(-24.67) | 18.39(-11.99) | 31.01^*^(-9.10) | 8.42(-0.37) | 13.02(-13.07) | -0.34(-6.51^*^) |
|  |  | 1h | 32.08(-26.96^*^) | 39.80 (-18.91) | 36.19^*^(-11.49) | 15.24(-8.92) | 12.76(-11.98) | 0.21(-4.65) |
|  |  | 2h | 44.05(-6.90) | 7.29(10.24) | 20.10(-7.91) | 7.01(4.62) | 9.64(-6.28) | 6.13^*^(-1.57) |
|  |  | 3h | 20.58(-12.53) | -13.99(15.95) | 10.18(-27.78^*^) | -8.18(18.70) | 2.35(-7.94) | 4.43(0.86) |

Abbreviations: HRV, heart rate variability; HR, heart rate; PM_2.5_, fine particulate matter; BC, black carbon; LF, low frequency power; HF, high frequency power; LF/HF, ratio of low–high frequency power; SDNN, standard deviation of all normal-to-normal (NN) intervals.

^*^ FDR-corrected p < 0.05; ^***^ FDR-corrected p < 0.01.

Table S4. Estimated percent changes (95% confidence intervals) in HRV and HR and p-values for interaction term in PM_2.5_-obesity per interquartile range increase in personal PM_2.5_ at 15-min moving average in the normal-weight and the obese groups during the waking and sleeping hours

| HRV indices | Normal-weight | Obese | p*-*value for the interaction term  (FDR corrected) |
| --- | --- | --- | --- |
| Waking hours |  |  |  |
| SDNN | 0.26(-2.47, 3.07) | 0.47(-2.12, 3.12) | 0.378 |
| Total power | 0.87(-4.84, 6.93) | 3.41(-3.51, 10.82) | 0.328 |
| HF | 4.05(-3.01, 11.62) | -13.31(-17.31, -9.12) | 0.017^*^ |
| LF | 0.65(-4.74, 6.34) | 2.19(-3.89, 8.66) | 0.189 |
| LF/HF | 4.66(-1.66, 11.39) | 13.50(7.49, 19.85) | 0.126 |
| HR | -2.54(-3.52, -1.54) | 2.70(1.44, 3.97) | < 0.001^*^ |
| Sleeping hours |  |  |  |
| SDNN | 12.14(-1.81, 28.06) | -1.67(-16.02, 15.15) | 0.114 |
| Total power | 23.76(-5.37, 61.85) | -26.77(-39.68, -11.10) | 0.133 |
| HF | 30.19(6.09, 59.76) | 7.72(-16.24, 38.53) | 0.040^*^ |
| LF | 24.14(-10.77, 72.71) | 6.58(-17.58, 37.81) | 0.186 |
| LF/HF | -0.78(-20.83, 24.33) | 13.01(-18.81, 57.30) | 0.237 |
| HR | 2.01(-2.13, 6.31) | -0.11(-3.79, 3.71) | 0.363 |

Abbreviations: HRV, heart rate variability; HR, heart rate; PM_2.5_, fine particulate matter; SDNN, standard deviation of all normal-to-normal (NN) intervals;

HF, high frequency power; LF, low frequency power; LF/HF, ratio of low–high frequency power.

^*^ FDR-corrected p < 0.05.

Table S5. Estimated percent changes (95% confidence intervals) in HRV and HR and p-values for interaction term in BC-obesity per interquartile range increase in personal BC at 15-min moving average in the normal-weight and the obese groups during the waking and sleeping hours

| HRV indices | Normal-weight | Obese | p-value for the interaction term  (FDR corrected) |
| --- | --- | --- | --- |
| Waking hours |  |  |  |
| SDNN | 2.90(-0.60, 6.53) | 0.79(-1.93, 3.60) | 0.215 |
| Total power | 1.95(-5.44, 9.92) | 1.03(-5.00, 7.43) | 0.347 |
| HF | 7.78(-1.17, 17.53) | -13.06(-17.43, -8.47) | 0.004^*^ |
| LF | -2.12(-9.70, 6.08) | -5.77(-10.73, -0.53) | 0.194 |
| LF/HF | 1.83(-6.06, 10.38) | 11.88(5.51, 18.65) | 0.086 |
| HR | -1.64(-3.22, -0.04) | 3.24(1.93, 4.56) | < 0.001^*^ |
| Sleeping hours |  |  |  |
| SDNN | 10.66(-3.14, 26.43) | -11.27(-22.27, 1.29) | 0.015^*^ |
| Total power | 28.47(-3.07, 70.27) | -30.54(-43.33, -14.86) | 0.014^*^ |
| HF | 14.26(-5.21, 37.72) | -22.48(-38.76, -1.87) | < 0.001^*^ |
| LF | 3.44(-20.93, 35.33) | -20.04(-35.23, -1.30) | 0.074 |
| LF/HF | 9.03(-12.85, 36.42) | 5.15(-21.68, 41.17) | 0.238 |
| HR | -0.01(-3.36, 3.46) | -7.09(-10.83, -3.19) | 0.267 |

Abbreviations: HRV, heart rate variability; HR, heart rate; BC, black carbon; SDNN, standard deviation of all normal-to-normal (NN) intervals;

HF, high frequency power; LF, low frequency power; LF/HF, ratio of low–high frequency power.

^*^ FDR-corrected p < 0.05.

Table S6. Estimated percent changes (95% confidence intervals) in HRV and HR and p-values for interaction term in PM_2.5_-obesity per interquartile range increase in personal PM_2.5_ at 30-min moving average in the normal-weight and the obese groups during the waking and sleeping hours

| HRV indices | Normal-weight | Obese | p-value for the interaction term  (FDR corrected) |
| --- | --- | --- | --- |
| Waking hours |  |  |  |
| SDNN | 0.13(-2.71, 3.06) | -0.13(-2.79, 2.61) | 0.326 |
| Total power | -0.01(-5.91, 6.26) | -1.27(-6.92, 4.73) | 0.282 |
| HF | 3.62(-3.73, 11.54) | -13.74(-17.83, -9.44) | 0.024^*^ |
| LF | -0.84(-6.36, 5.01) | -2.53(-7.44, 2.63) | 0.343 |
| LF/HF | 3.82(-2.69, 10.78) | 14.01(7.81, 20.57) | 0.111 |
| HR | -1.94(-3.18, -0.68) | 2.57(1.25, 3.90) | < 0.001^*^ |
| Sleeping hours |  |  |  |
| SDNN | 8.46(-4.79, 23.55) | -2.03(-16.70, 15.22) | 0.128 |
| Total power | 22.09(-7.83, 61.71) | -2.95(-29.03, 32.72) | 0.144 |
| HF | 32.04(7.97, 61.48) | 11.74(-14.34, 45.77) | 0.054 |
| LF | 24.97(-10.51, 74.52) | 15.21(-16.12, 58.22) | 0.191 |
| LF/HF | -1.76(-21.85, 23.50) | 17.00(-12.25, 56.00) | 0.268 |
| HR | 3.03(-1.30, 7.54) | -1.37(-5.16, 2.57) | 0.130 |

Abbreviations: HRV, heart rate variability; HR, heart rate; PM_2.5_, fine particulate matter; SDNN, standard deviation of all normal-to-normal (NN) intervals;

HF, high frequency power; LF, low frequency power; LF/HF, ratio of low–high frequency power.

^*^ FDR-corrected p < 0.05.

Table S7. Estimated percent changes (95% confidence intervals) in HRV and HR and p-values for interaction term in BC-obesity per interquartile range increase in personal BC at 30-min moving average in the normal-weight and the obese groups during the waking and sleeping hours

| HRV indices | Normal-weight | Obese | p-value for the interaction term  (FDR corrected) |
| --- | --- | --- | --- |
| Waking hours |  |  |  |
| SDNN | 4.08(0.43, 7.87) | 3.14(-0.26, 6.66) | 0.183 |
| Total power | 4.18(-3.62, 12.60) | 1.10(-5.34, 7.98) | 0.279 |
| HF | 16.69(7.99, 26.08) | -5.71(-11.64, 0.61) | < 0.001^*^ |
| LF | -0.81(-7.82, 6.75) | -3.58(-8.99, 2.16) | 0.292 |
| LF/HF | 0.24(-7.75, 8.92) | 15.02(8.03, 22.46) | 0.022^*^ |
| HR | -2.11(-3.78, -0.41) | 3.45(2.01, 4.90) | < 0.001^*^ |
| Sleeping hours |  |  |  |
| SDNN | 13.02(-1.83, 30.11) | -13.07(-23.85, -0.75) | 0.011^*^ |
| Total power | 27.34(-4.81, 70.34) | -31.19(-44.05, -15.37) | 0.007^*^ |
| HF | 34.37(10.49, 63.41) | -8.05(-27.68, 16.92) | 0.004^*^ |
| LF | 11.07(-8.91, 35.44) | -8.59(-26.21, 13.23) | 0.025 ^*^ |
| LF/HF | 3.31(-16.32, 27.55) | -0.66(-25.41, 32.31) | 0.386 |
| HR | -0.80(-4.33, 2.87) | -5.03(-8.69, -1.24) | 0.111 |

Abbreviations: HRV, heart rate variability; HR, heart rate; BC, black carbon; SDNN, standard deviation of all normal-to-normal (NN) intervals;

HF, high frequency power; LF, low frequency power; LF/HF, ratio of low–high frequency power.

^*^ FDR-corrected p < 0.05.

Table S8. Estimated percent changes (95% confidence intervals) in HRV and HR and p-values for interaction term in PM_2.5_-obesity per interquartile range increase in personal PM_2.5_ at 1-h moving average in the normal-weight and the obese groups during the waking and sleeping hours

| HRV indices | Normal-weight | Obese | p-value for the interaction term  (FDR corrected) |
| --- | --- | --- | --- |
| Waking hours |  |  |  |
| SDNN | 0.64(-2.48, 3.86) | -0.31(-3.15, 2.60) | 0.259 |
| Total power | 0.24(-6.20, 7.12) | -1.89(-7.83, 4.43) | 0.247 |
| HF | -4.43(-10.59, 2.16) | -15.59(-19.83, -11.13) | 0.012^*^ |
| LF | -1.47(-7.64, 5.10) | 2.07(-4.64, 9.25) | 0.233 |
| LF/HF | 2.67(-4.32, 10.17) | 16.53(9.82, 23.65) | 0.039^*^ |
| HR | -1.39(-2.81, 0.06) | 3.00(1.54, 4.47) | < 0.001^*^ |
| Sleeping hours |  |  |  |
| SDNN | 10.57(-4.33, 27.78) | -3.61(-18.76, 14.37) | 0.137 |
| Total power | 22.11(-8.87, 63.64) | -7.24(-34.00, 30.36) | 0.116 |
| HF | 26.48(5.42, 51.75) | 25.84(-5.48, 67.53) | 0.115 |
| LF | 0.90(-21.78, 30.15) | 8.31(-21.66, 49.73) | 0.153 |
| LF/HF | 0.76(-19.72, 26.47) | 9.21(-25.38, 59.84) | 0.367 |
| HR | 7.31(2.52, 12.32) | -2.88(-6.98, 1.41) | 0.004^*^ |

Abbreviations: HRV, heart rate variability; HR, heart rate; PM_2.5_, fine particulate matter; SDNN, standard deviation of all normal-to-normal (NN) intervals;

HF, high frequency power; LF, low frequency power; LF/HF, ratio of low–high frequency power.

^*^ FDR-corrected p < 0.05.

Table S9. Estimated percent changes (95% confidence intervals) in HRV and HR and p-values for interaction term in BC-obesity per interquartile range increase in personal BC at 1-h moving average in the normal-weight and the obese groups during the waking and sleeping hours

| HRV indices | Normal-weight | Obese | p-value for the interaction term  (FDR corrected) |
| --- | --- | --- | --- |
| Waking hours |  |  |  |
| SDNN | 6.49(2.63, 10.50) | 2.34(-0.85, 5.64) | 0.102 |
| Total power | 8.53(0.13, 17.63) | 4.38(-2.62, 11.88) | 0.258 |
| HF | 23.13(13.7, 33.34) | -14.91(-19.74, -9.78) | < 0.001^*^ |
| LF | 2.21(-5.45, 10.49) | -0.43(-6.33, 5.83) | 0.308 |
| LF/HF | 0.08(-8.16, 9.06) | 20.84(11.91, 30.47) | 0.007^*^ |
| HR | -1.95(-3.81, -0.06) | 2.90(1.33, 4.50) | < 0.001^*^ |
| Sleeping hours |  |  |  |
| SDNN | 12.76(-3.71, 32.05) | -11.98(-23.06, 0.7) | 0.018^*^ |
| Total power | 32.49(-1.01, 77.32) | -26.39(-43.35, -4.36) | 0.007^*^ |
| HF | 40.88(14.82, 72.87) | -9.19(-28.57, 15.45) | < 0.001^*^ |
| LF | 34.06(-2.77, 84.83) | -6.04(-26.45, 20.02) | 0.007^*^ |
| LF/HF | 14.56(-14.59, 53.65) | -3.92(-26.62, 25.79) | 0.197 |
| HR | -0.72(-4.52, 3.24) | -3.16(-7.09, 0.94) | 0.103 |

Abbreviations: HRV, heart rate variability; HR, heart rate; BC, black carbon; SDNN, standard deviation of all normal-to-normal (NN) intervals;

HF, high frequency power; LF, low frequency power; LF/HF, ratio of low–high frequency power.

^*^ FDR-corrected p < 0.05.

Table S10. Estimated percent changes (95% confidence intervals) in HRV and HR and p-values for interaction term in PM_2.5_-obesity per interquartile range increase in personal PM_2.5_ at 3-h moving average in the normal-weight and the obese groups during the waking and sleeping hours

| HRV indices | Normal-weight | Obese | p-value for the interaction term  (FDR corrected) |
| --- | --- | --- | --- |
| Waking hours |  |  |  |
| SDNN | 2.21(-2.25, 6.88) | 2.63(-2.42, 7.94) | 0.364 |
| Total power | 7.45(-2.16, 18.02) | 2.6(-6.18, 12.20) | 0.154 |
| HF | -1.67(-10.55, 8.09) | -5.92(-14.48, 3.50) | 0.037^*^ |
| LF | 5.18(-3.88, 15.09) | 8.31(-1.62, 19.25) | 0.310 |
| LF/HF | 8.54(-2.06, 20.28) | 24.79(14.70, 35.75) | 0.110 |
| HR | -1.65(-4.32, 1.09) | 1.88(-0.32, 4.13) | 0.175 |
| Sleeping hours |  |  |  |
| SDNN | 1.30(-12.05, 16.69) | -11.50(-22.63, 1.24) | 0.335 |
| Total power | 4.66(-17.11, 32.14) | -24.53(-38.18, -7.88) | 0.215 |
| HF | 20.26(4.68, 38.16) | 58.09(15.90, 115.63) | 0.248 |
| LF | -2.85(-30.63, 36.07) | 18.84(-12.95, 62.22) | 0.190 |
| LF/HF | -10.61(-27.12, 9.65) | 10.78(-24.23, 61.96) | 0.251 |
| HR | 7.52(3.23, 12.00) | -5.75(-9.69, -1.64) | < 0.001^*^ |

Abbreviations: HRV, heart rate variability; HR, heart rate; PM_2.5_, fine particulate matter; SDNN, standard deviation of all normal-to-normal (NN) intervals;

HF, high frequency power; LF, low frequency power; LF/HF, ratio of low–high frequency power.

^*^ FDR-corrected p < 0.05.

Table S11. Estimated percent changes (95% confidence intervals) in HRV and HR and p-values for interaction term in BC-obesity per interquartile range increase in personal BC at 3-h moving average in the normal-weight and the obese groups during the waking and sleeping hours

| HRV indices | Normal-weight | Obese | p-value for the interaction term  (FDR corrected) |
| --- | --- | --- | --- |
| Waking hours |  |  |  |
| SDNN | 6.99(1.50, 12.78) | 3.21(-3.09, 9.91) | 0.038^*^ |
| Total power | 18.65(5.32, 33.67) | -5.00(-14.43, 5.47) | 0.017^*^ |
| HF | 30.25(15.76, 46.55) | -19.37(-27.88, -9.85) | < 0.001^*^ |
| LF | 2.31(-8.76, 14.72) | 18.97(5.99, 33.54) | 0.127 |
| LF/HF | -7.10(-19.12, 6.71) | 47.08(30.04, 66.35) | < 0.001 |
| HR | -5.38(-8.08, -2.60) | 5.32(2.51, 8.21) | < 0.001^*^ |
| Sleeping hours |  |  |  |
| SDNN | 2.35(-15.45, 23.89) | -7.94(-19.29, 5.02) | 0.155 |
| Total power | 17.82(-15.28, 63.87) | -8.85(-30.25, 19.11) | 0.062 |
| HF | 13.32(-8.94, 41.03) | -29.73(-43.82, -12.12) | 0.015^*^ |
| LF | -8.72(-38.14, 34.69) | 19.78(-12.95, 64.80) | 0.250 |
| LF/HF | -9.82(-27.82, 12.66) | 16.49(-15.84, 61.25) | 0.230 |
| HR | 2.40(-1.88, 6.86) | 0.49(-3.65, 4.82) | 0.388 |

Abbreviations: HRV, heart rate variability; HR, heart rate; BC, black carbon; SDNN, standard deviation of all normal-to-normal (NN) intervals;

HF, high frequency power; LF, low frequency power; LF/HF, ratio of low–high frequency power.

^*^ FDR-corrected p < 0.05.

Table S12. Estimated percent changes in HRV and HR per interquartile range increase in ambient PM_2.5_ and BC at different moving averages in the normal-weight (obese) group throughout the day

| Pollutant | Averaging period | HRV | | | | | HR |
| --- | --- | --- | --- | --- | --- | --- | --- |
|  |  | Total power | LF | HF | LF/HF | SDNN |  |
| PM_2.5_ | 1h | 4.26(-13.00) | -0.79(-10.80) | 6.66(-9.68) | 10.05(7.63) | -3.96(-4.55) | -2.94(0.89) |
|  | 2h | 0.41(-12.87) | 6.63(-10.93) | 14.62(12.08) | 13.28(7.77) | -0.07(-3.50) | -1.94(-1.47) |
|  | 3h | 2.41(-10.00) | 27.98(-8.28) | 10.36(12.81) | 13.39(6.43) | -1.54(-2.34) | -1.26(-2.63) |
| BC | 1h | -4.74(-5.75) | 4.94(-3.64) | -1.85(15.25) | 17.26(-10.08) | -4.98(-0.85) | 0.69(-3.42^*^) |
|  | 2h | -4.50(-3.86) | 14.01(-4.05) | -13.23(12.50) | 10.76(-11.13) | -5.10(-0.95) | 0.95(-4.50^***^) |
|  | 3h | -4.71(-1.39) | 4.15(-0.89) | -2.38(18.50) | 15.44(-10.87) | -5.28(-0.29) | -0.10(-4.28^***^) |

Abbreviations: HRV, heart rate variability; HR, heart rate; PM_2.5_, fine particulate matter; BC, black carbon; LF, low frequency power; HF, high frequency power;

LF/HF, ratio of low–high frequency power; SDNN, standard deviation of all normal-to-normal (NN) intervals.

^*^ FDR-corrected p < 0.05; ^***^ FDR-corrected p < 0.01.

Table S13. Estimated percent changes in HRV and HR per interquartile range increase in ambient PM_2.5_ and BC at different moving averages in the normal-weight (obese) group during the waking and sleeping hours

|  | Pollutant | Averaging period | HRV | | | | | HR |
| --- | --- | --- | --- | --- | --- | --- | --- | --- |
|  |  |  | Total power | LF | HF | LF/HF | SDNN |  |
| Waking hours | PM_2.5_ | 1h | 0.63(-5.18) | -1.86(-6.31) | -6.70(5.84) | -2.36(-0.90) | 0.54(1.24) | -5.25^*^ (-3.40^*^) |
|  |  | 2h | 10.06(-4.88) | 7.17(-6.31) | 11.75(7.84) | -3.58(-3.16) | 2.11(2.47) | -5.73^*^ (-3.73^*^) |
|  |  | 3h | 8.87(-0.92) | 10.54(-2.15) | 23.18(8.17) | -4.99(3.29) | -1.92(3.67) | -6.09^*^ (-1.99^*^) |
|  | BC | 1h | -0.20(3.67) | 4.94(6.49) | 18.00(7.25) | 8.29(4.58) | -2.83(0.76) | -1.52(-1.97^*^) |
|  |  | 2h | 6.10(6.31) | 13.46(8.15) | -4.38(7.51) | 5.71(4.20) | -0.86(1.24) | -3.07^*^ (-2.14^*^) |
|  |  | 3h | 7.63(9.25) | 15.70(13.18) | 1.13(7.91) | 7.39(7.25) | -0.17(1.72) | -4.55^*^ (-1.50^*^) |
| Sleeping hours | PM_2.5_ | 1h | 5.38(5.00) | -0.50(-2.22) | 12.83(6.80) | -3.61(9.82) | -3.66(-5.36) | -3.43(-1.17) |
|  |  | 2h | 8.51(3.89) | 6.59(-0.67) | 6.40(-2.35) | 6.72(12.49) | -5.62(-1.89) | -0.91(-0.24) |
|  |  | 3h | 11.51(1.73) | 13.44(-2.26) | 5.42(-5.24) | 12.24(8.72) | -0.60(-0.65) | 1.06(1.58) |
|  | BC | 1h | 12.72(-21.52) | 34.40(-19.75) | -7.98(-14.33) | 29.55(-14.09) | -3.13(-8.74) | 6.69(-0.12) |
|  |  | 2h | 7.69(-19.56) | 20.21(-22.59) | -5.05(-10.41) | 15.01(-17.28) | -5.46(-9.15) | 6.49(-0.96) |
|  |  | 3h | 9.08(-17.83) | 11.79(-22.13) | -9.71(-8.74) | 17.49(-15.12) | -7.35(-9.94) | 7.89(-1.46) |

Abbreviations: HRV, heart rate variability; HR, heart rate; PM_2.5_, fine particulate matter; BC, black carbon; LF, low frequency power; HF, high frequency power; LF/HF, ratio of low–high frequency power; SDNN, standard deviation of all normal-to-normal (NN) intervals.

^*^ FDR-corrected p < 0.05


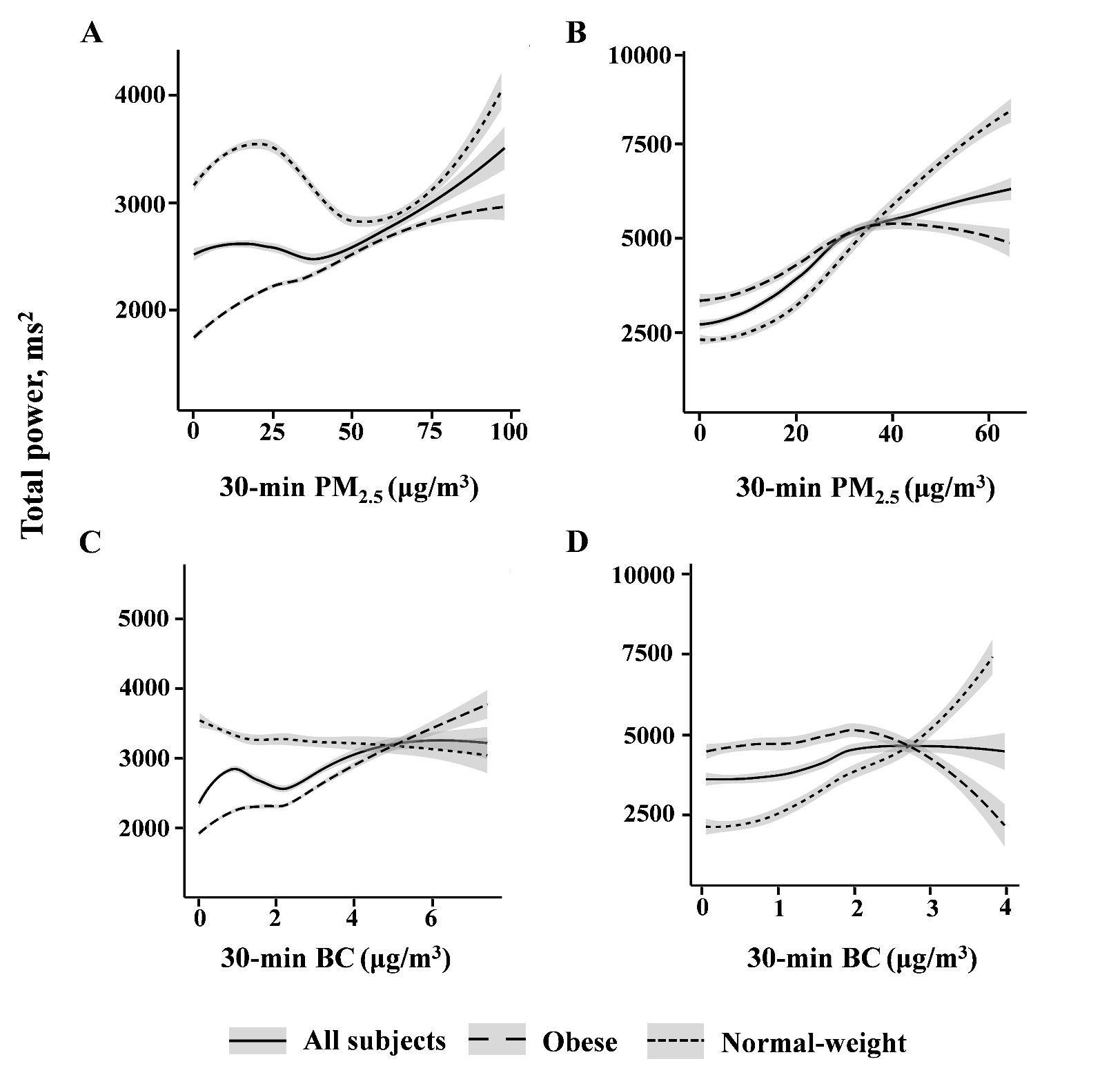


**Figure S1.** Exposure–response relationship between 30-min personal PM_2.5_ and 30-min personal BC moving average and total power with 95% confidence intervals under generalized additive models in all subjects, normal-weight and obese individuals. The degree of freedom was estimated by generalized cross validation. A and C, waking hours; B and D, sleeping hours.

Abbreviations: PM_2.5_, fine particulate matter; BC, black carbon.


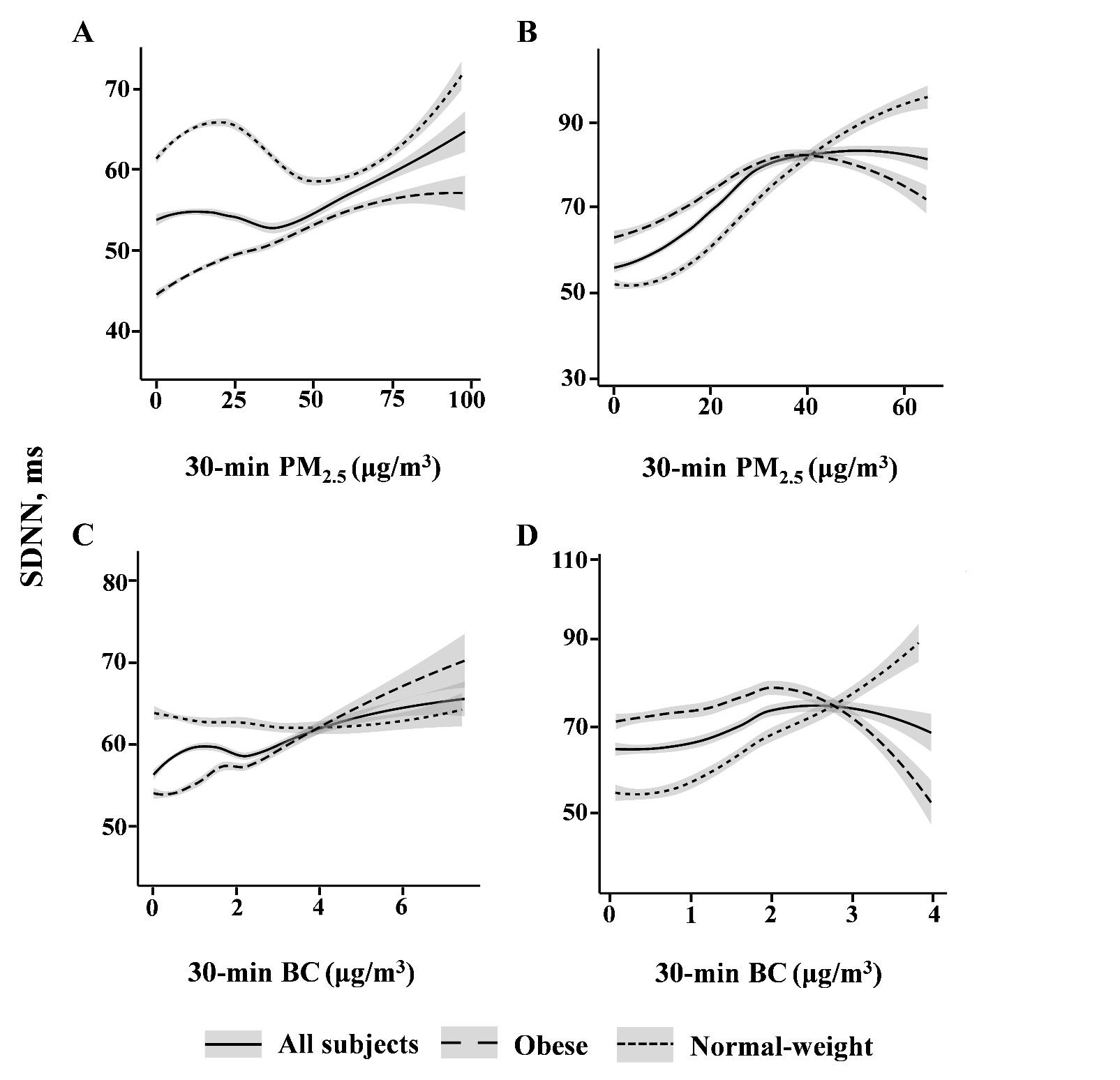
Figure S2. Exposure–response relationship between 30-min personal PM_2.5_ and 30-min personal BC moving average and SDNN with 95% confidence intervals under generalized additive models in all subjects, normal-weight and obese individuals. The degree of freedom was estimated by generalized cross validation. A and C, waking hours; B and D, sleeping hours.

Abbreviations: PM_2.5_, fine particulate matter; BC, black carbon; SDNN, standard deviation of all normal-to-normal (NN) intervals.


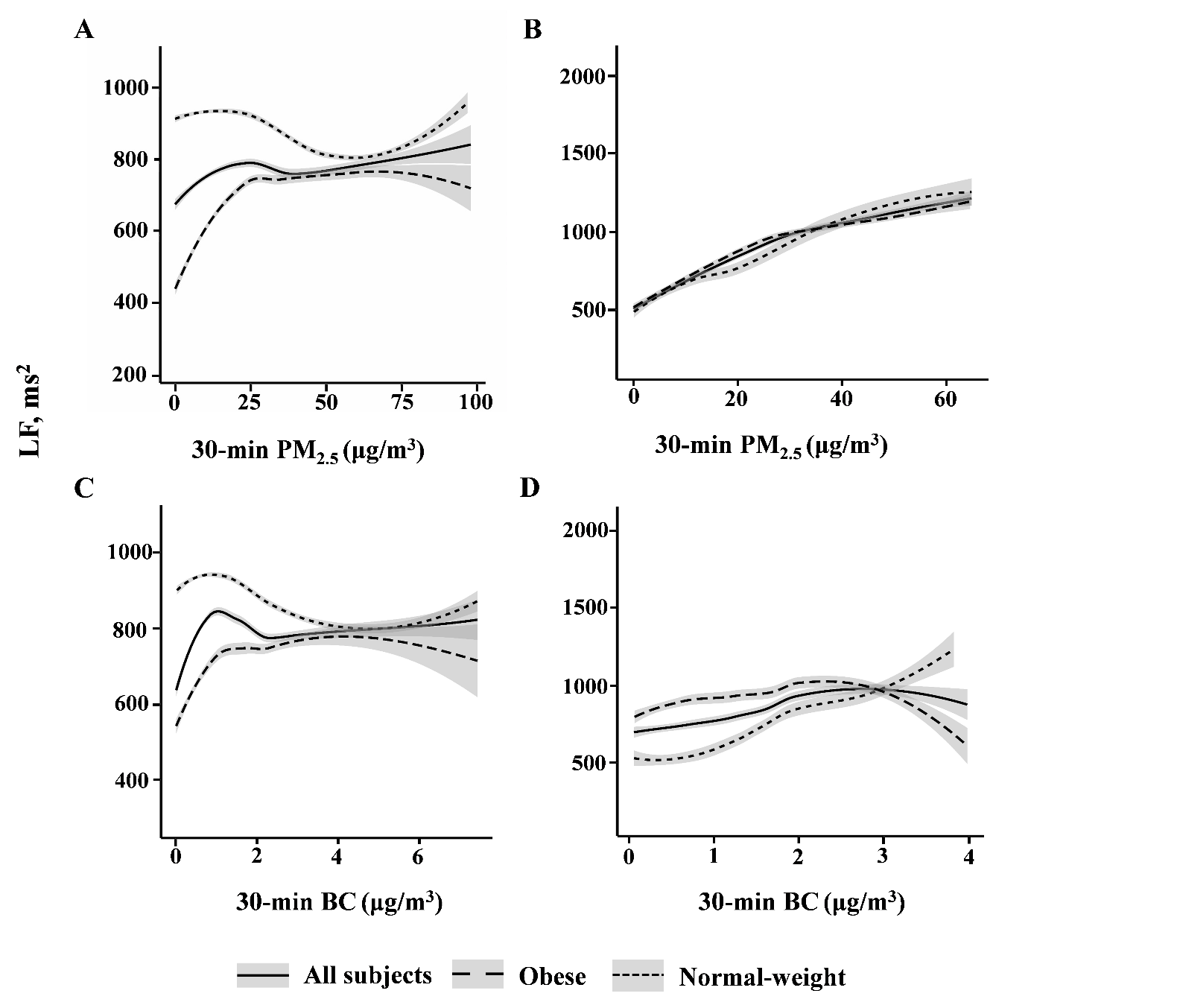
Figure S3. Exposure–response relationship between 30-min personal PM_2.5_ and 30-min personal BC moving average and LF with 95% confidence intervals under generalized additive models in all subjects, normal-weight and obese individuals. The degree of freedom was estimated by generalized cross validation. A and C, waking hours; B and D, sleeping hours.

Abbreviations: PM_2.5_, fine particulate matter; BC, black carbon; LF, low frequency power.


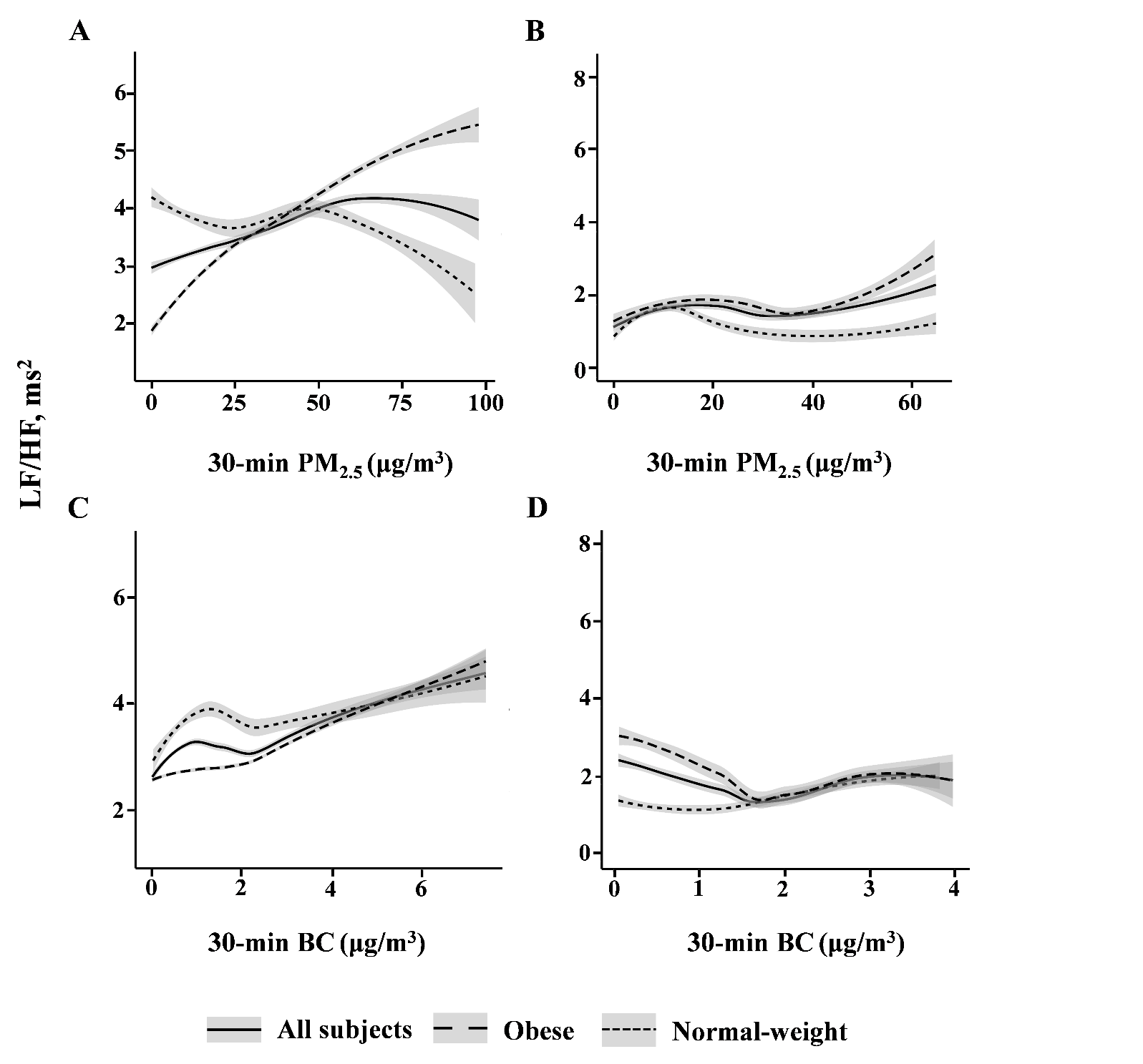
Figure S4. Exposure–response relationship between 30-min personal PM_2.5_ and 30-min personal BC moving average and LF/HF with 95% confidence intervals under generalized additive models in all subjects, normal-weight and obese individuals. The degree of freedom was estimated by generalized cross validation. A and C, waking hours; B and D, sleeping hours.

Abbreviations: PM_2.5_, fine particulate matter; BC, black carbon; LF/HF, ratio of low–high frequency power.


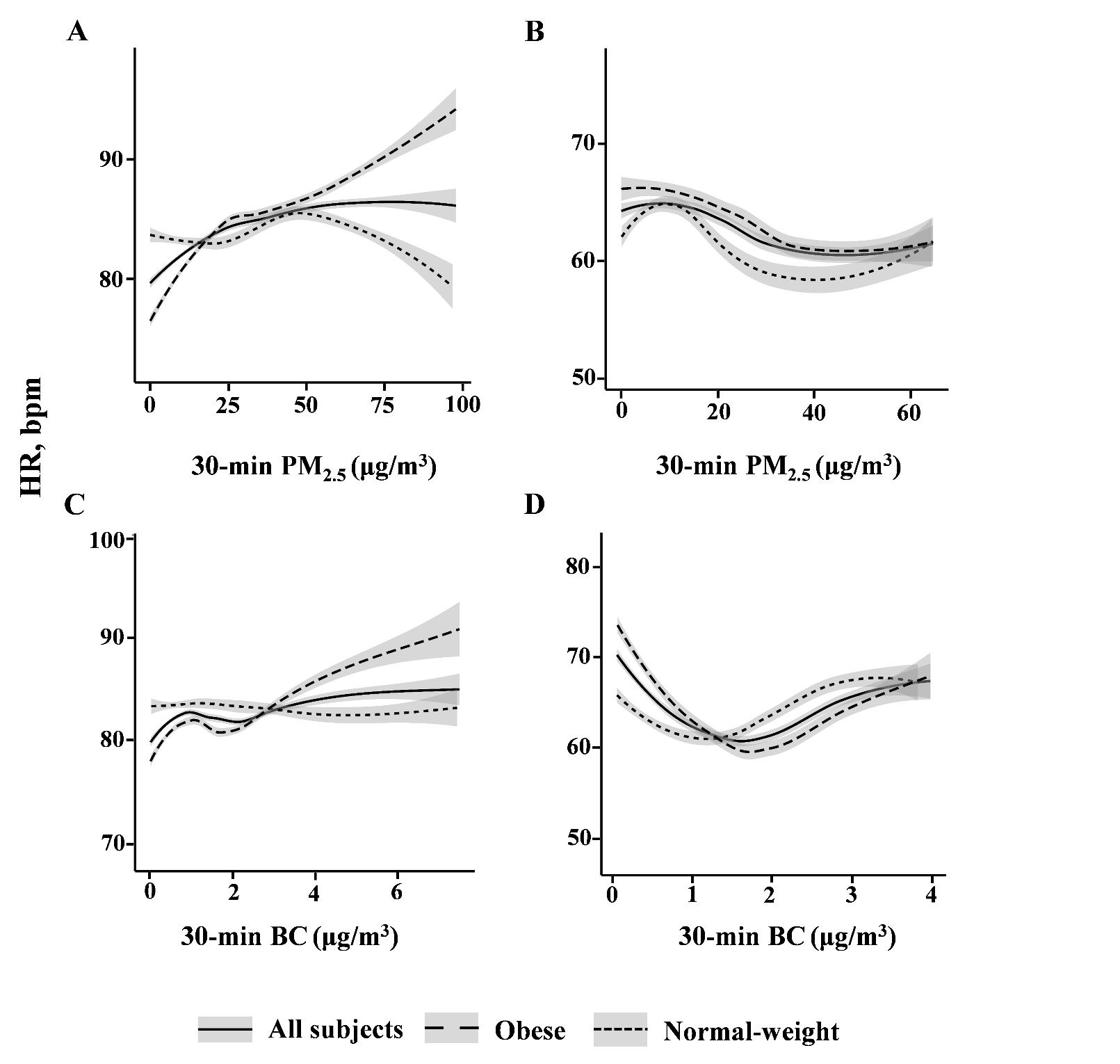
Figure S5. Exposure–response relationship between 30-min personal PM_2.5_ and 30-min personal BC moving average and HR with 95% confidence intervals under generalized additive models in all subjects, normal-weight and obese individuals. The degree of freedom was estimated by generalized cross validation. A and C, waking hours; B and D, sleeping hours.

Abbreviations: PM_2.5_, fine particulate matter; BC, black carbon; HR, heart rate; bpm, beat per minute.
